# A universal ligand mediated method for large scale synthesis of transition metal single atom catalysts

**DOI:** 10.1038/s41467-019-12510-0

**Published:** 2019-10-08

**Authors:** Hongzhou Yang, Lu Shang, Qinghua Zhang, Run Shi, Geoffrey I. N. Waterhouse, Lin Gu, Tierui Zhang

**Affiliations:** 1 Key Laboratory of Photochemical Conversion and Optoelectronic Materials Technical Institute of Physics and Chemistry, Chinese Academy of Sciences, 100190 Beijing, China; 2 Center of Materials Science and Optoelectronics Engineering, University of Chinese Academy of Sciences, 100049 Beijing, China; 3 Beijing National Laboratory for Condensed Matter Physics, Institute of Physics, Chinese Academy of Sciences, 100190 Beijing, China; 4 School of Chemical Sciences, The University of Auckland, Auckland, 1142 New Zealand; 5 Songshan Lake Materials Laboratory, 523808 Dongguan, China

**Keywords:** Materials chemistry, Electrocatalysis, Nanoscale materials

## Abstract

There is interest in metal single atom catalysts due to their remarkable activity and stability. However, the synthesis of metal single atom catalysts remains somewhat ad hoc, with no universal strategy yet reported that allows their generic synthesis. Herein, we report a universal synthetic strategy that allows the synthesis of transition metal single atom catalysts containing Cr, Mn, Fe, Co, Ni, Cu, Zn, Ru, Pt or combinations thereof. Aberration-corrected high-angle annular dark-field scanning transmission electron microscopy and extended X-ray absorption fine structure spectroscopy confirm that the transition metal atoms are uniformly dispersed over a carbon black support. The introduced synthetic method allows the production of carbon-supported metal single atom catalysts in large quantities (>1 kg scale) with high metal loadings. A Ni single atom catalyst exhibits outstanding activity for electrochemical reduction of carbon dioxide to carbon monoxide, achieving a 98.9% Faradaic efficiency at −1.2 V.

## Abstract (web summary)

Single-atom catalysts are a promising class of catalytic materials, but general synthetic methods are limited. Here, the authors develop a ligand-mediated strategy that allows the large-scale synthesis of diverse transition metal single atom catalysts supported on carbon.

## Introduction

Homogeneous and heterogeneous catalysts are widely used across chemical industry^1–3^. Homogeneous catalysts offer high efficiency and high selectivity due to their easily accessible active sites and maximal active metal utilization but suffer from low stability and poor recyclability. In contrast, heterogeneous catalysts offer excellent stability and good recyclability, but their active metal utilization is typically low (only surface atoms in supported metal nanoparticles actually participate in catalytic reactions)^4,5^. Single-atom catalysts (SACs) have recently emerged as a promising new class of catalytic materials, capturing the inherent advantages of both homogeneous and heterogeneous catalysts. SACs typically comprise individual metal atoms (M) immobilized on a support, with many SACs being stabilized through porphyrin-like MN_4_ surface coordination geometries^6^. Accordingly, SACs have the dual advantage of nearly 100% atomic utilization (similar to homogeneous catalysts) as well as high stability and easy separation from reaction media (features of heterogeneous catalysts). Furthermore, when the size of active metal component is reduced from the nanometer lengths (i.e., nanoparticles or clusters) to the atomic level (i.e., SACs), new electronic states of the metal can appear thereby imparting unique performance^7^.

Following the pioneering discovery of SACs by Zhang et al.^8^, numerous experimental and theoretical studies have highlighted the outstanding catalytic potential of SACs for hydrogenation reactions^9^, carbon monoxide (CO) oxidation^10^, methane conversion^11^, carbon dioxide (CO_2_) reduction^12–16^, hydrogen evolution^17^, oxygen reduction^18^, dinitrogen (N_2_) reduction^19^, and other chemical transformations^1^. Various synthetic strategies have been used to access SACs, including physical and chemical methods. Physical methods, such as mass-selected soft landing^20^ and atomic layer deposition^21^, are not especially amenable for large-scale production of SACs since they require complex and expensive equipment. Chemical strategies involving the atomic dispersion of metals on a catalyst support, typically through the adsorption of metal precursors followed by reduction and stabilization, are therefore more practical^22^. Another common chemical approach toward SACs involves the carbonization of zeolitic imidazolate frameworks containing Co (ZIF-67) and Zn (ZIF-8)^23,24^. Although the feasibility of these two synthetic approaches has been confirmed in a number of studies^25^, the low yields and low metal loadings (~1 wt.%) of these approaches limit their practical usefulness. For many practical applications, SACs with high metal loadings are demanded. Further, existing chemical strategies are not very versatile and cannot easily be adapted to synthesize SACs containing other transition metals. Some recent studies have attempted to develop more universal synthetic methods toward SACs. Duan et al. used holey graphene as substrate to synthesize Fe, Co, and Ni-SACs^26^. Qu et al. reported that Cu, Co, or Ni-SACs could be fabricated on a carbon support containing abundant defect sites (derived by pyrolysis of ZIF-8) using a gas-migration method^27^. However, a single synthetic strategy that allows the large-scale synthesis of SACs containing almost any transition metal with high metal loadings has proven elusive. Such a synthetic strategy would expedite the utilization of SACs across the chemical sector. Recently, Beller and co-workers^28^ reported a novel protocol for preparing nanoscale cobalt-based catalysts via pyrolysis of organometallic amine complexes on activated carbon. The coordination of the metal cations by ligands during the pyrolysis step was found to reduce Co agglomeration leading to the formation of ultrafine supported Co nanoparticles. However, the high temperature (800 °C) used in that study was an obstacle to obtaining SACs. By lowering the pyrolysis temperature, SACs should be accessible since the kinetics and thermodynamic driving force for metal nanoparticle formation should be greatly reduced, motivating a detailed investigation.

**Fig. 1:**
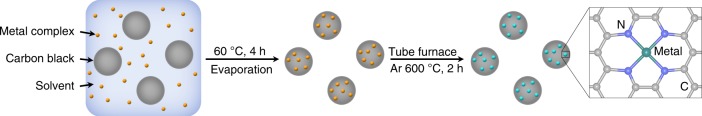
The universal synthesis procedure. Metal single-atom catalysts (M-SACs) are prepared in two steps

Herein we report the successful synthesis of a library of M-SACs (M = Ni, Mn, Fe, Co, Cr, Cu, Zn, Ru, Pt, and combinations thereof) by complexing metal cations with 1,10-phenanthroline, adsorbing the resulting metal complexes onto commercial carbon black (Ketjenblack EC-300J), followed by pyrolysis of the resulting surface-modified carbons at 600 °C under an argon atmosphere (Fig. 1, see “Methods” section for full details). Aberration-corrected high-angle annular dark-field scanning transmission electron microscopy (HAADF-STEM) and extended X-ray absorption fine structure (EXAFS) spectroscopy confirm that all M-SACs contain metals atomically dispersed in porphyrin-like MN_4_ sites over the carbon support (no cluster or nanoparticle formation is observed). X-ray absorption near edge spectroscopy (XANES) and X-ray photoelectron spectroscopy (XPS) reveal that all M-SACs contained M^2+^ ions. Further, compositional analysis by inductively coupled plasma optical emission spectroscopy (ICP-OES) establish that the synthesis method could produce M-SACs with high metal loadings (easily up to 1.8 wt.% for all metals, Supplementary Table 1). The performance of the different M-SACs are subsequently evaluated for electrochemical CO_2_ reduction, with the Ni-SAC containing 2.5 wt.% demonstrating outstanding performance for CO_2_ reduction to CO (~99% Faradaic efficiency at −1.2 V).

## Results

### Characterization of M-SACs

**Fig. 2:**
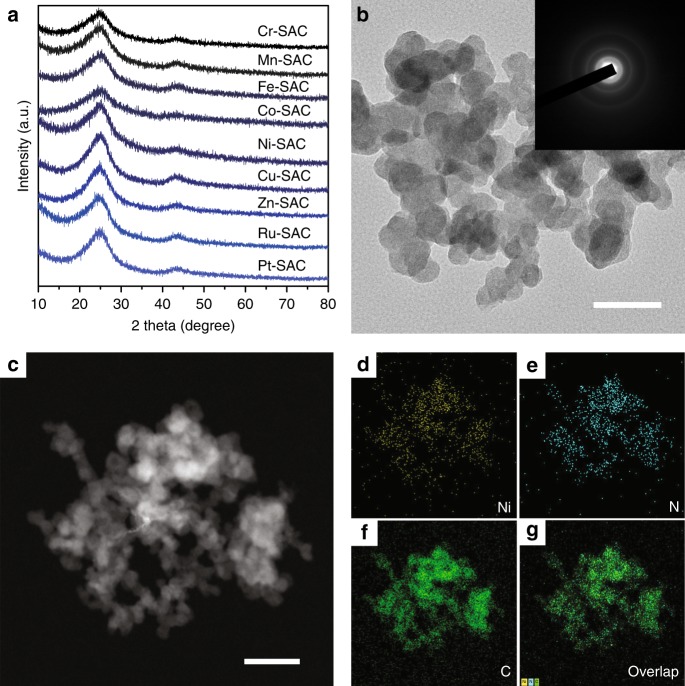
Characterization of metal single-atom catalysts. **a** XRD patterns for different metal single-atom catalysts. **b** TEM image and SAED pattern (inner) for Ni-SAC-2.5, scale bar 100 nm. **c** STEM image for Ni-SAC-2.5, scale bar 100 nm. **d**–**g** EDX maps for Ni-SAC-2.5

The structure of the M-SACs synthesized using the new “ligand-mediated” method (M = Ni, Mn, Fe, Co, Cr, Cu, Zn, Ru, Pt, and combinations thereof) were first probed by powder X-ray diffraction (XRD) and transmission electron microscopy (TEM). The XRD patterns of all M-SACs showed broad peaks centered around 24° and 44° (Fig. 2a), corresponding to the (002) and (100) planes of graphite. No metal-related peaks were observed in the XRD patterns. Conversely, pyrolysis of the Ni-SAC precursor at 800 °C resulted in the formation of some Ni clusters (Supplementary Fig. 1), in agreement with Beller’s results^28^. It can be concluded that the lower pyrolysis temperature of 600 °C used in the current work prevented metal nanocluster formation. TEM images for the M-SACs confirmed that no metal clusters or nanoparticles were present on the carbon black support (Fig. 2b and Supplementary Figs. 2–9). Energy-dispersive X-ray (EDX) element maps (Fig. 2c–g) for Ni-SAC confirmed a very homogeneous dispersion of Ni and N over the carbon support. Selected-area electron diffraction (Fig. 2b, inset) showed that the Ni-SAC possessed low crystallinity and no Ni nanoparticles (evidenced by an absence of any intense rings), consistent with the XRD results. The Raman spectrum for Ni-SAC (Supplementary Fig. 10) contained two peaks at 1359 and 1595 cm^−1^, which can readily be assigned to disordered sp^3^ carbon (D band) and graphitic sp^2^ carbon (G band), respectively. The *I*_D_/*I*_G_ ratio for Ni-SAC (0.87) was lower than that determined for pristine carbon black (1.01), indicating that the graphitic content of the carbon support increased after the pyrolysis step used to synthesize Ni-SAC.

**Fig. 3:**
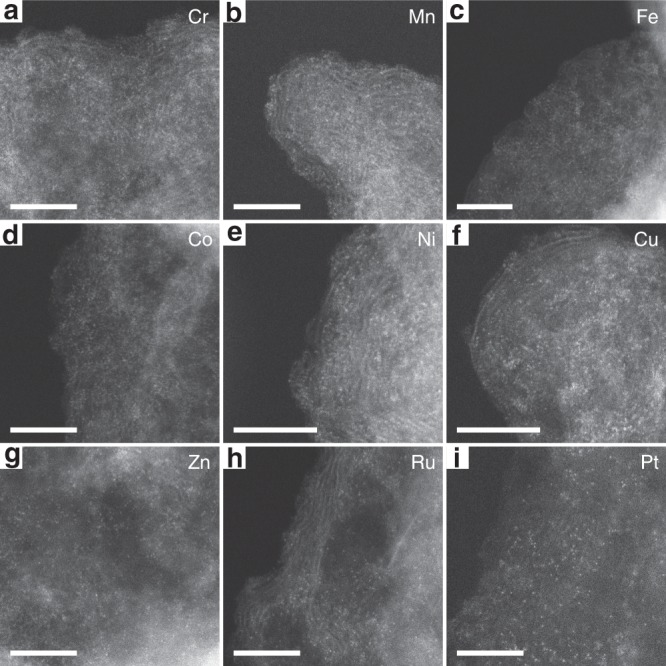
Images for various metal single-atom catalysts. Aberration-corrected high-angle annular dark-field scanning transmission electron microscopy for **a** Cr-SAC, **b** Mn-SAC, **c** Fe-SAC, **d** Co-SAC, **e** Ni-SAC, **f** Cu-SAC, **g** Zn-SAC, **h** Ru-SAC, and **i** Pt-SAC. Scale bar: 5 nm

HAADF-STEM was used to definitively confirm the atomic dispersion of metal atoms over the carbon support in the different M-SACs. As shown in the HAADF-STEM images of the SACs (Fig. 3a–g), the first row of transition metals (Cr, Mn, Fe, Co, Ni, Cu, Zn) appeared as high-density bright dots, indicating that these metals existed in the single-atom form on the carbon substrate. Similar results were found for the SACs containing the second-row (Ru) and third-row (Pt) transition metals (Fig. 3h, i, respectively), where Ru and Pt atoms were found to be homogeneously dispersed as single atoms on the carbon support. A further advantage of the synthetic strategy reported herein is that it allows the synthesis of multicomponent metal SACs, which are very difficult to obtain via conventional pyrolysis methods used to synthesize SACs. For example, bimetallic Fe/Co-SACs could be successfully synthesized. XRD (Supplementary Fig. 11) and HAADF-STEM (Supplementary Fig. 12) confirmed that Fe and Co atoms were atomically dispersed over the carbon support. Ru/Fe-SACs, Ru/Co-SACs, and Ru/Ni-SACs were also synthesized. XRD patterns for these catalysts (Supplementary Fig. 13) contained two broad peaks corresponding to the carbon black support and no other features. These studies highlight the universality of the new synthesis method for the preparation of M-SACs.

**Fig. 4:**
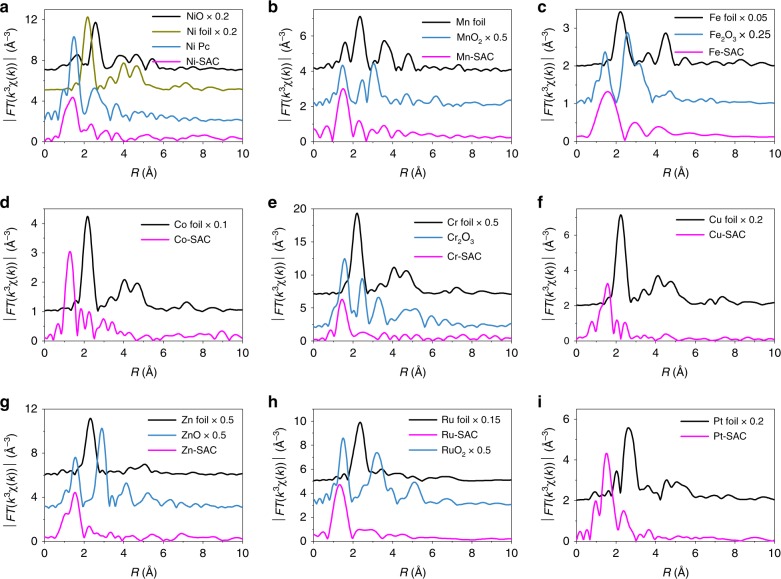
Metal *K*-edge extended X-ray absorption fine structure (*R* space plots). **a** Ni-SAC, **b** Mn-SAC, **c** Fe-SAC, **d** Co-SAC, **e** Cr-SAC, **f** Cu-SAC, **g** Zn-SAC, **h** Ru-SAC, and **i** Pt-SAC. In all the M-SACs, the metal atoms existed as isolated M^2+^ centers in a MN_4_ coordination environment

EXAFS spectroscopy is very sensitive to the local environment of metal atoms. Accordingly, EXAFS was applied to confirm the presence of single metal atom sites in the various M-SACs. Figure 4 shows metal *K*-edge EXAFS data (*R* space plots) for the SACs along with data for relevant reference samples. For each M-SAC, the *R* space plots were quite distinct from that of the corresponding metal or metal oxide reference. Ni-SAC showed an intense peak at approximately 1.45 Å, corresponding to the first Ni-N coordination shell, with the oscillation being very similar to that observed for nickel phthalocyanine (Ni Pc), indicating that Ni atoms in the Ni-SAC had a similar local environment with that of Ni atoms in Ni Pc. Fitting result determined that Ni in Ni-SAC was coordinated fourfold by N atoms, as is also found in Ni Pc (Supplementary Fig. 14). The other M-SACs also showed only a single peak in *R* space (at shorter lengths than the typical M–M distance in the corresponding metal foil), confirming that no metal–metal bonds existed in the M-SACs. These results were in good accord with the findings of the HAADF-STEM analyses. XANES spectra and *k*^3^-weighted *K*-space spectra for the different M-SACs are shown in Supplementary Figs. 15–32. For all nine M-SAC samples, the adsorption edge was higher than that of the corresponding metal foil, indicating that M-SACs contained metal atoms in cationic states (likely the M^2+^ state)^29–31^.

The chemical composition and elemental states of the M-SACs were investigated by XPS. The Ni 2p XPS spectrum for Ni-SAC (Supplementary Fig. 33a) showed a Ni 2p_3/2_ peak at 855.6 eV, higher than that observed for Ni^0^ and slightly lower than that for Ni^2+^ in NiO. The data provide strong evidence for the presence of Ni^2+^ in the Ni-SACs, with the slightly lower binding energy compared to NiO due to coordination by N rather than O. The C 1s spectrum (Supplementary Fig. 33b) was deconvoluted into two peaks, corresponding to C-C or C=C (neutral carbon and adventitious hydrocarbons) and C-N species. The N 1s spectrum (Supplementary Fig. 33c) for the Ni-SAC could be deconvoluted into three peaks, corresponding to pyridinic-N, pyrrolic-N, and graphitic-N. The presence of N, which originated from the 1,10-phenanthroline ligand, adds further weight to the proposal that the metal atoms in the M-SACs existed in MN_4_ coordination environments (as established from the EXAFS analyses). XPS data collected for the other M-SACs (Supplementary Figs. 34–41) were similar to that reported for the Ni-SAC, with the metal core-level spectra providing strong evidence for the presence of M^2+^ single-atom states.

**Fig. 5:**
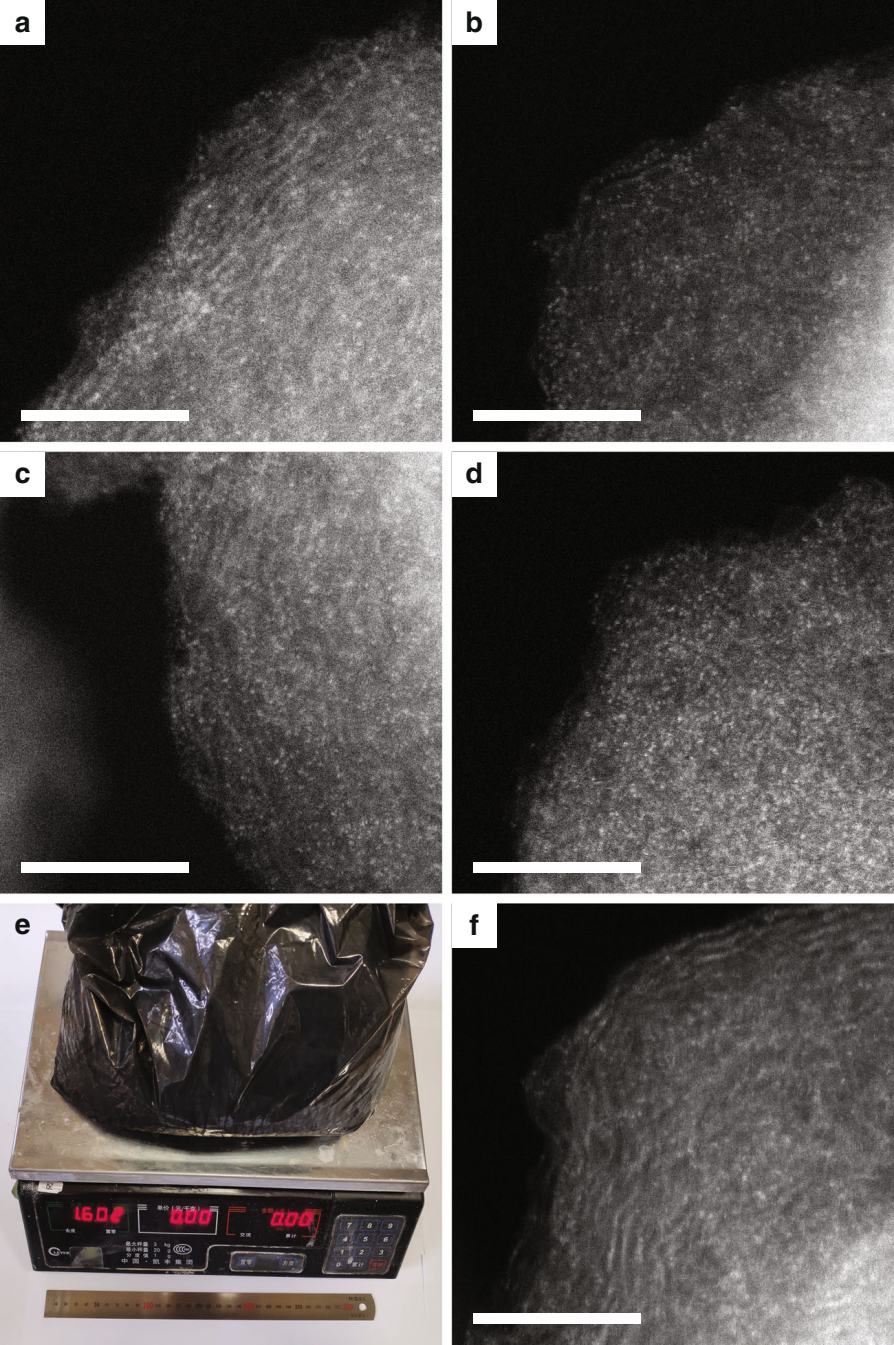
Characterization and large-scale synthesis of Ni catalysts with different loadings. **a** HAADF-STEM image for Ni-SAC with 2.5 wt.% Ni loading. **b** HAADF-STEM image for Ni-SAC with 3.4 wt.% Ni loading. **c** HAADF-STEM image for Ni-SAC with 4.5 wt.% Ni loading. **d** HAADF-STEM image for Ni-SAC with 5.3 wt.% Ni loading. Scale bar: 5 nm. **e** Photograph for Ni-SAC-2.5 synthesized in large scale. **f** HAADF-STEM image for Ni-SAC-2.5 synthesized in large scale. Scale bar: 5 nm

Synthesizing SACs with high metal loadings and in large quantities has traditionally proved technically challenging and remains an obstacle to the widespread utilization of SACs. In traditional synthesis methods, SACs are obtained by high-temperature pyrolysis of metal organic frameworks (MOFs) or metal-impregnated graphitic carbon nitride (g-C_3_N_4_). Such methods produce single-atom sites, though a large fraction of metal atoms in the precursor are typically transformed to metal clusters or nanoparticles. Here a low pyrolysis temperature of only 600 °C was used to synthesize the M-SACs, which was possible since we used a commercial carbon as the support (thus higher temperatures were not needed to obtain a partially graphitic and conductive carbon support as is the case with MOFs or g-C_3_N_4_ precursors). Accordingly, the method we developed was expected to be more amenable for the synthesis of M-SACs with high metal loadings. To test this hypothesis, we synthesized a series of Ni-SACs with different Ni loadings (denoted as Ni-SAC-*x*, where *x* is the actual Ni loading in wt.%), with the actual Ni loadings determined by ICP-OES. Using our new “ligand-mediated” synthetic strategy, Ni-SACs with Ni loadings of 2.5, 3.4, 4.5, and 5.3 wt.% were obtained, with the high loadings being superior to those of most other Ni-SACs reported to date (Supplementary Table 2). Supplementary Figs. 42–45 show TEM images for the Ni-SACs prepared at the different Ni loadings. For all Ni-SAC-*x* samples, no metal nanoparticles were observed. The XRD patterns for each sample contained broad peaks at 24° and 44° (Supplementary Fig. 46) corresponding to the (002) and (100) planes of graphite. To confirm that Ni was atomically dispersed at each loading, HAADF-STEM and EXAFS analyses were performed. Figure 5a–d show HAADF-STEM images for the Ni-SAC-*x* samples. For all samples, the atoms were atomically dispersed, with a few very small Ni clusters seen for the Ni-SAC-5.3 sample. Supplementary Figs. 47–49 show Ni *K*-edge EXAFS *R* space plots, Ni *K*-edge XANES, and Ni *K*-edge EXAFS *K*-space plots, respectively, for the Ni-SACs synthesized with different Ni loadings. The XANES data confirmed that all the Ni-SACs contained Ni^2+^. The *R* space plots showed a distinct feature at ~1.45 Å, corresponding to Ni-N first coordination shell. Results confirm that all of the Ni-SAC-*x* samples contained Ni^2+^ atoms fourfold coordinated by N. In addition, since our method was based on the modification of a commercially available carbon, it is suitable for scaling up. To prove this, we performed some large-scale syntheses, producing up to 1.6 kg of Ni-SAC in a single synthesis (Fig. 5e, with potential for further production scale-up). No metallic nickel peaks were seen in the XRD pattern of the large batch sample (Supplementary Fig. 50) or corresponding HAADF-STEM image (Fig. 5f), indicating that nickel was atomically dispersed over the carbon black.

### Electroreduction of CO_2_ to CO

**Fig. 6:**
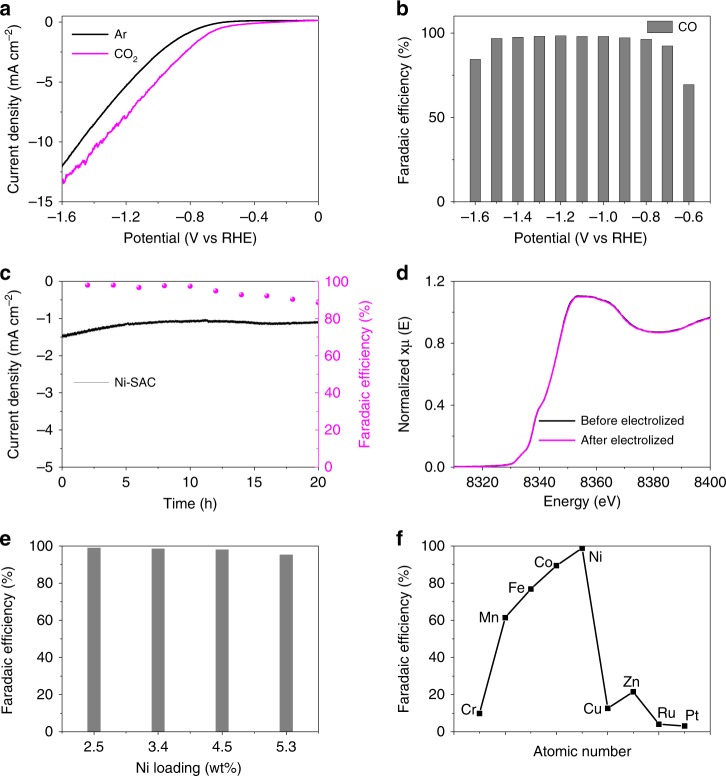
CO_2_ electrochemical reduction performance of metal single-atom catalysts. **a** Cathodic LSV scans for Ni-SAC-2.5 in Ar-saturated and CO_2_-saturated aqueous 0.1 M KHCO_3_ solutions. **b** FE_CO_ for Ni-SAC-2.5 at different potentials in a CO_2_-saturated aqueous 0.1 M KHCO_3_ solution. **c** Stability test for Ni-SAC-2.5 at −0.8 V vs RHE. **d** XANES spectra for Ni-SAC-2.5 before and after CO_2_RR tests. **e** FE_CO_ for Ni-SAC-x at −1.2 V vs RHE. **f** FE_CO_ for M-SACs at −1.2 V vs RHE

Phthalocyanines and porphyrins, in which metal ions are surrounded fourfold by N, are important families of enzyme-mimicking catalysts. Metal porphyrins linked by organic struts to form covalent organic frameworks are particularly active for the CO_2_ reduction reaction (CO_2_RR), a key reaction for high-density renewable energy storage and CO_2_ capture^32^. Our characterization studies above revealed that the local environment of metal atoms in the M-SACs were very similar to that of the same metals in phthalocyanines and porphyrins. Further, here the SACs were dispersed on a carbon support with good electrical conductivity, which was expected to greatly enhance electrocatalytic performance. This motivated a detailed study of the performance of the various M-SACs synthesized in the current work for electrocatalytic CO_2_RR. The electrocatalytic CO_2_RR activities of the different M-SACs were measured in a sealed H-type cell, with gas chromatography used for product detection. Figure 6a shows the linear sweep voltammetry (LSV) curves for Ni-SAC in Ar and CO_2_ saturated electrolytes. The current density was much higher in the CO_2_-saturated electrolyte, indicating that Ni-SAC was more active for CO_2_ reduction than hydrogen evolution. Figure 6b shows the Faradaic efficiency to CO (FE_CO_) for Ni-SAC at different potentials. Ni-SAC displayed excellent CO_2_RR performance, maintaining a high FE_CO_ (above 90%) over a wide range of potentials (from −0.7 to −1.5 V vs reversible hydrogen electrode (RHE)) and a low Faradaic efficiency to H_2_ (Supplementary Fig. 51), with the highest FE_CO_ (98.9%) achieved at −1.2 V. No liquid products were detected in the aqueous phase by nuclear magnetic resonance spectroscopy (Supplementary Fig. 52), confirming a very high selectivity to CO. The FE_CO_ was higher than most state-of-the-art catalysts (Supplementary Table 2). We also tested the performance of Ni-SAC for the CO_2_RR at a fixed potential (−0.8 V vs RHE). As shown in Fig. 6c, only a very slight decrease in the current density occurred over 20 h. Figure 6d shows Ni *K*-edge XANES spectra for the Ni-SAC before and after CO_2_RR tests. The before and after spectra were almost identical, confirming that the Ni-SAC was very stable under the conditions of CO_2_RR.

The performance of the Ni-SACs with different Ni loadings, Ni-SAC synthesized in large scale (1.6 kg), and M-SACs with different metals were also evaluated for CO_2_RR. The FE_CO_ for the Ni-SACs with Ni loadings of 2.5, 3.4, 4.5, and 5.3 wt.% at −1.2 V were 98.9%, 98.5%, 98.0%, and 95.3%, respectively (Fig. 6e). The FE_CO_ of the Ni-SAC-5.3 sample was a bit smaller than values determined for the other samples with lower Ni loadings, which may have been due to the co-existence of Ni-SACs and tiny Ni clusters in this sample. To demonstrate the scalability of the “ligand-mediated” method, we also test the CO_2_RR performance of the Ni-SAC synthesized in large scale (~1.6 kg). The catalytic properties of the large batch sample were almost identical to those of samples synthesized on a 70-mg scale (Supplementary Fig. 53), confirming the scalability of the new synthetic method. Furthermore, thanks to the universality of the “ligand-mediated” method, it was possible to quantitatively compare the effect of central metal atom in M-SACs on CO_2_RR performance. The CO_2_RR performance of the M-SACs, each with similar metal loadings, were tested at −1.2 V vs RHE (Fig. 6f). Interestingly, the FE_CO_ of the M-SACs plotted against metal atomic number followed a volcano curve, with the Ni-SAC displaying the highest activity for CO_2_RR. On transitioning from Cr to Ni, the FE_CO_ increased, whereas on going from Ni to Zn the FE_CO_ decreased abruptly. The observation of a “volcano curve” likely originates from the d-band center position of each metal in the M-SACs, with the d-band center position of Ni^2+^ in the Ni-SACs being most ideal for CO_2_RR^33,34^.

## Discussion

As mentioned above, a wide range of M-SACs (M = Ni, Mn, Fe, Co, Cr, Cu, Zn, Ru, Pt, and combinations thereof) were successfully prepared using a single “ligand-mediated” synthetic strategy. In this strategy, metal complexes (i.e., M^2+^ ions coordinated by 1,10-phenanthroline ligands) facilitate the creation of “porphyrin-like” single metal atom sites on the carbon black surface, thereby tightly binding the metal cations and preventing the aggregation of the metal atoms into clusters or nanoparticles. Further, the direct use of a conductive carbon support circumvents the need to use high pyrolysis temperatures that are obviously detrimental to SACs’ stability (typically temperatures as high as 900 °C are needed to produce carbon materials with good conductivities if derived from ZIF precursors). HAADF-STEM and EXAFS analysis indicated that the metal atoms were atomically dispersed (as M^2+^) in all the M-SACs. The developed synthetic method thus confers the following advantages over existing methods for SAC fabrication: (1) it is universal and can be applied to synthesize various transition metal SACs and even bimetallic SACs; (2) commercially available, conductive carbons can be used as a support, which is advantageous for electrocatalytic applications; (3) the method allows large-scale SAC production (kilogram scale, with potential for further production scale-up); and (4) the method yields SACs with high metal loadings. Therefore, this work represents a totally new paradigm for the synthesis of SACs.

In summary, we have developed a universal and robust “ligand-mediated” method for the synthesis of M-SACs with high metal contents. The method can be used to synthesize M-SACs containing first-, second- and third-row transition metals on carbon supports. Ni-SACs synthesized by the new method show excellent activity and stability for electrochemical CO_2_ reduction to CO (FE_CO_ of 98.9% at −1.2 V at the optimum Ni loading of 2.5 wt.%). As well as M-SACs containing single metals (Cr, Mn, Fe, Co, Cu, Zn, Ru, Pt), the synthetic method could also be used to synthesize bimetallic SACs. Results pave the way for the large-scale fabrication of M-SACs with high metal loadings for CO_2_ reduction, oxygen reduction or evolution, hydrogen evolution, N_2_ reduction, and other chemical reactions.

## Methods

### Chemicals

Nickel (II) acetate tetrahydrate and manganese (II) acetate tetrahydrate were purchased from Shanghai Macklin Biochemical Technology Co., Ltd. Iron (II) acetate was purchased from J&K Scientific., Ltd. Cobalt (II) acetate tetrahydrate was purchased from Guangdong GHTECH Co., Ltd. Chromium (III) nitrate nonahydrate and copper (II) acetate monohydrate were purchased from Xilong Scientific Co., Ltd. Ruthenium (III) chloride was purchased from Innochem Co., Ltd. Platinum (II) chloride was purchased from Acros Organic Co., Ltd. 1,10-Phenanthroline monohydrate was purchased from Sinopharm Chemical Reagent Co., Ltd. Zinc (II) acetate dihydrate, ethanol, and dimethyl sulfoxide were purchased from Beijing Chemical Works. All reagents and solvents were of analytical grade and used as received without additional purification. The CO_2_ and Ar feed gases were purchased from Beijing SIDADE RM Science and Technology Co., Ltd.

### Synthesis of Ni-SAC-*x* (where *x* is the wt.% Ni)

For the synthesis of Ni-SAC-2.5% (i.e., a Ni SAC containing 2.5 wt.% Ni), nickel (II) acetate tetrahydrate (12.4 mg) and 1,10-phenanthroline monohydrate (29.7 mg) were dissolved in 2 mL of ethanol followed by stirring for approximately 20 min at room temperature. Subsequently, carbon black (69.6 mg) was added into the solution, and the resulting solution was heated in an oil-bath at 60 °C for 4 h under continuous magnetic stirring. The resulting dispersion was then heated at 80 °C in air for 12 h to evaporate the ethanol, yielding a black solid. The black solid obtained was lightly ground with a mortar and pestle, then transferred into a ceramic crucible and placed in a tube furnace. The black solid was then heated to 600 °C at a rate of 10 °C min^−1^ under an argon atmosphere and then held at 600 °C for 2 h. The product obtained after cooling to room temperature was denoted as Ni-SAC-2.5%. The Ni-SAC-3.4%, Ni-SAC-4.5%, and Ni-SAC-5.3% samples were obtained using a similar procedure, except that the amount of nickel (II) acetate tetrahydrate used was increased to 18.7, 24.9, and 49.7 mg, respectively, and the amount of 1,10-phenanthroline monohydrate used was increased proportionally to 44.6, 59.5, and 118.9 mg, respectively, to maintain a 1,10-phenanthroline:Ni molar ratio of 3.

### Synthesis of Mn-SAC, Fe-SAC, Co-SAC, and Zn-SAC

The Mn-SAC, Fe-SAC, Co-SAC, and Zn-SAC samples were prepared using a similar procedure to that described above for Ni-SAC-2.5, except that the amount of 1,10-phenanthroline monohydrate and metal salt were adjusted as required to achieve a 1,10-phenanthroline:M molar ratio of 3. The amounts of metal salt and 1,10-phenanthroline monohydrate used in each synthesis were: manganese (II) acetate tetrahydrate (19.8 mg) and 1,10-phenanthroline monohydrate (101.9 mg); iron (II) acetate (13.8 mg) and 1,10-phenanthroline monohydrate (100.3 mg); cobalt (II) acetate tetrahydrate (18.8 mg) and 1,10-phenanthroline monohydrate (94.99 mg); and zinc (II) acetate dihydrate (14.9 mg) and 1,10-phenanthroline monohydrate (85.6 mg).

### Synthesis of Cr-SAC, Cu-SAC, Ru-SAC, and Pt-SAC

Cr-SAC, Cu-SAC, Ru-SAC, and Pt-SAC were prepared using a similar procedure to that described for Ni-SAC-2.5, except that dimethyl sulfoxide rather than ethanol was used as the solvent. The amounts of metal salt and 1,10-phenanthroline monohydrate used in each synthesis were: chromium (III) nitrate nonahydrate (34.2 mg) and 1,10-phenanthroline monohydrate (107.7 mg); copper (II) acetate monohydrate (13.9 mg) and 1,10-phenanthroline monohydrate (88.1 mg); ruthenium (III) chloride (9.1 mg) and 1,10-phenanthroline monohydrate (55.4 mg); and platinum (II) chloride (6.1 mg) and 1,10-phenanthroline monohydrate (28.7 mg). Owing to the higher boiling point of dimethyl sulfoxide, the solvent evaporation step (that preceded sample heating in the tube furnace) was performed at 190 °C instead of 80 °C.

### Synthesis of bimetallic SACs

Fe/Co-SAC was prepared using a similar procedure to that described for Ni-SAC-2.5, except that the amount of metal salt was adjusted to iron (II) acetate (13.8 mg) and cobalt (II) acetate tetrahydrate (18.8 mg), and the amount of 1,10-phenanthroline monohydrate was adjusted to 195.29 mg. Ru/Fe-SAC, Ru/Co-SAC, and Ru/Ni-SAC were prepared using a similar procedure to that described for Ni-SAC-2.5, except that the dimethyl sulfoxide rather than ethanol was used as the solvent. The amounts of metal salt and 1,10-phenanthroline monohydrate used in each synthesis were: ruthenium (III) chloride (9.1 mg), iron (II) acetate (13.8 mg), and 1,10-phenanthroline monohydrate (155.7 mg); ruthenium (III) chloride (9.1 mg), cobalt (II) acetate tetrahydrate (18.8 mg), and 1,10-phenanthroline monohydrate (150.39 mg); and ruthenium (III) chloride (9.1 mg), nickel (II) acetate tetrahydrate (12.4 mg), and 1,10-phenanthroline monohydrate (85.1 mg).

### Synthesis of Ni-SAC on a large scale

For the synthesis of Ni-SAC on a large scale, nickel (II) acetate tetrahydrate (165.0 g) and 1,10-phenanthroline monohydrate (920.7 g) were dissolved in 2.5 L of ethanol followed by stirring at room temperature. Subsequently, carbon black (924.0 g) was added into the solution, and the resulting solution was then heated at 60 °C for 4 h under continuous stirring. The resulting dispersion was then heated at 80 °C in air for 12 h to evaporate ethanol, yielding a black solid that was ground to a powder. The black powder was then heated to 600 °C at a rate of 10 °C min^−1^ under an argon atmosphere, and then held at 600 °C for 2 h. The product was obtained after cooling to room temperature.

### Catalyst characterization

TEM images were collected on a HITACHI HT-7700 (HITACHI, Japan) microscope operating at an accelerating voltage of 100 kV. HAADF-STEM images and EDX elemental maps were obtained on an ARM-200CF (JEOL, Tokyo, Japan) operating at 200 kV and equipped with double spherical aberration correctors. The resolution of the probe defined by the objective pre-field was 78 pm. XRD patterns were obtained on a Bruker D8 Focus X-ray diffractometer equipped with Cu Kα radiation source (*λ* = 1.5405 Å) operating at 40 kV. XPS data were collected on a VGESCALABMKII X-ray photoelectron spectrometer using a non-monochromatized Al-Kα X-ray source (hν = 1486.6 eV). XAFS data were obtained at the Beijing Synchrotron Radiation Facility (1W1B). Raman spectra were collected on a Renishaw inVia-Reflex spectrometer system and excited by a 532-nm laser. The metal loadings in the SACs were measured by ICP-OES (Varian 710).

### Electrochemical measurements

A three-electrode sealed H-type cell was used in all electrochemical tests. Catalysts (4 mg) were dispersed in a solution of H_2_O (0.5 mL), ethanol (0.48 mL), and 5 wt.% Nafion solution (0.02 mL). After sonication for 2 h, a uniform ink-like dispersion was obtained. Next, 2 μL of the dispersion was dropped onto a polished glassy carbon electrode (GCE), and the resulting electrode was allowed to dry in air for several hours. This catalyst/GCE served as the working electrode in subsequent electrochemical tests. An Ag/AgCl electrode and a Pt wire were used as the reference electrode and the counter electrode, respectively. LSV data were collected at a scan rate of 10 mV s^−1^ in a 0.1 M KHCO_3_ electrolyte. When testing the Faradaic efficiency, the working electrode was held at a constant potential for 30 min. The gas products evolved were detected by a Shimadzu GC-2014 chromatograph (Shimadzu Co., Japan) equipped with a HP PLOT Al_2_O_3_ column and a flame ionization detector.

## Supplementary information

Supplementary Information

## Data Availability

All relevant data are available from the corresponding author on request.
