# Supplementary material for: A universal ligand mediated method for large scale synthesis of transition metal single atom catalysts
Source: Nat Commun. 2019 Oct 8;10:4585. doi: 10.1038/s41467-019-12510-0 (PMC6783464; doi:10.1038/s41467-019-12510-0)
Supplement: Supplementary file 1 — Supplementary Information [file 41467_2019_12510_MOESM1_ESM.pdf]

Supplementary Information

A universal ligand mediated method for large scale synthesis of transition metal single atom catalysts

Yang et al.

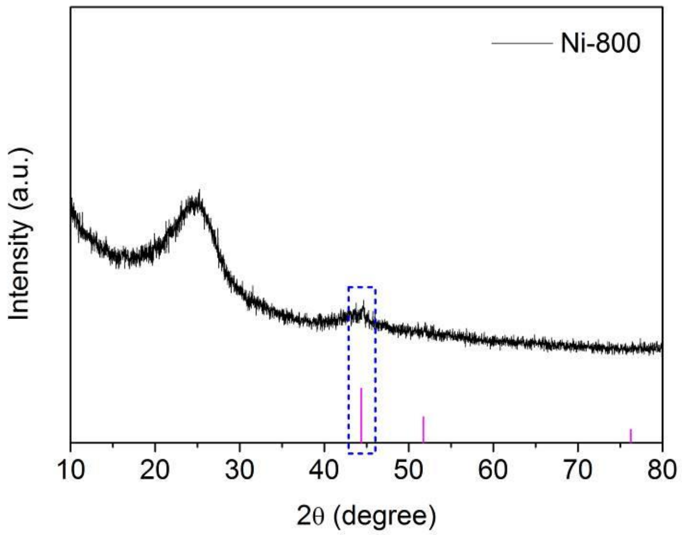

Supplementary Figure 1 | Characterization of the Ni precursor following pyrolysis at 800 ^{o}C. XRD pattern for Ni-800.

Supplementary Figure 1 confirmed that some Ni clusters were formed when the Ni precursor was pyrolyzed at 800 ^{o}C. Clearly at such temperatures, Ni atoms have sufficient mobility to aggregate into small clusters and nanoparticles.

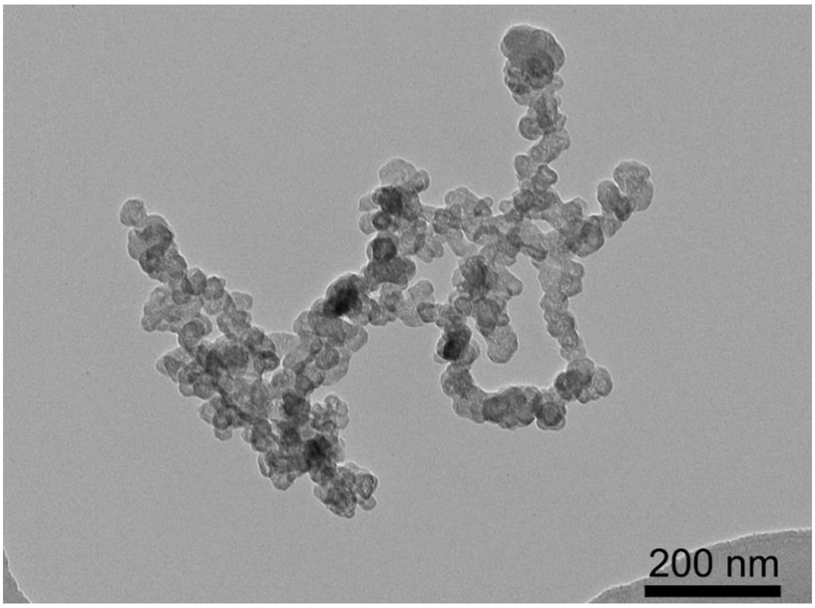

Supplementary Figure 2 | Morphology of Cr-SAC. TEM image of Cr-SAC.

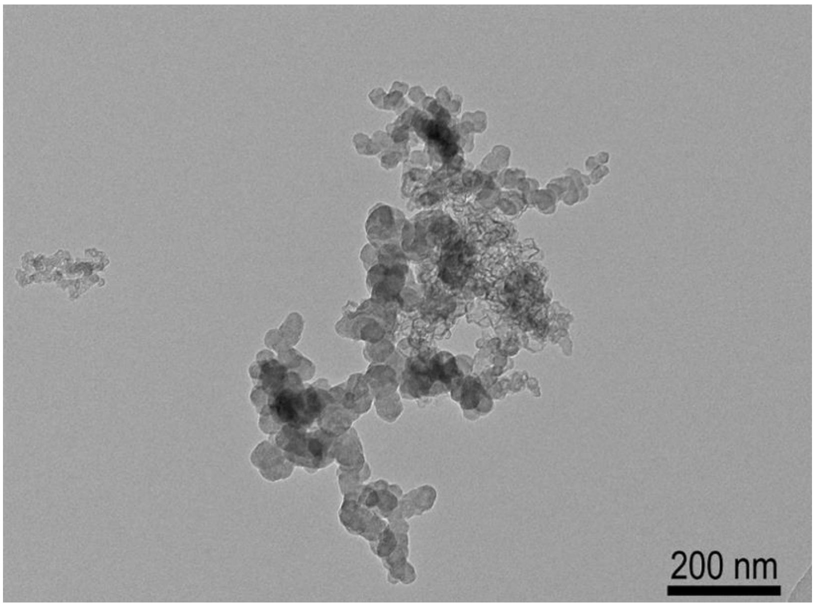

Supplementary Figure 3 | Morphology of Mn-SAC. TEM image of Mn-SAC.

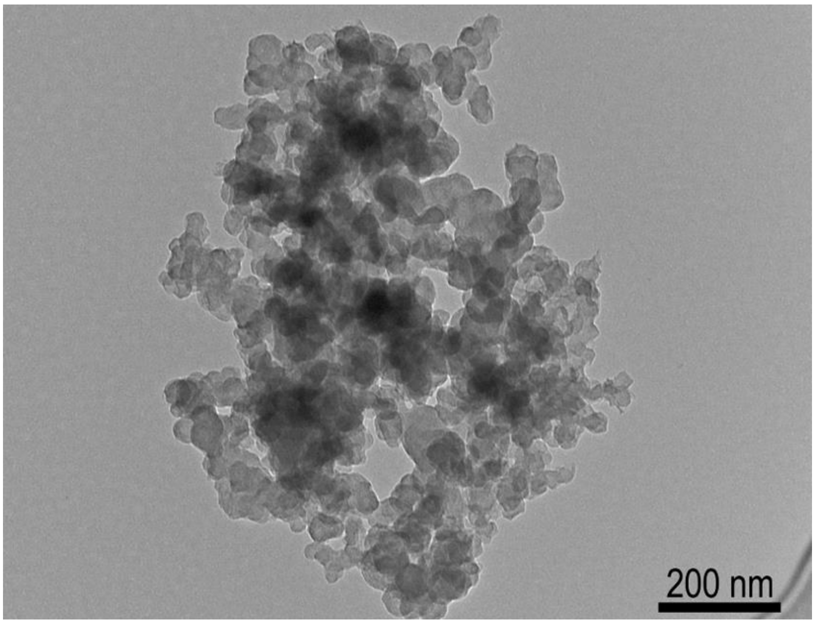

Supplementary Figure 4 | Morphology of Fe-SAC. TEM image of Fe-SAC.

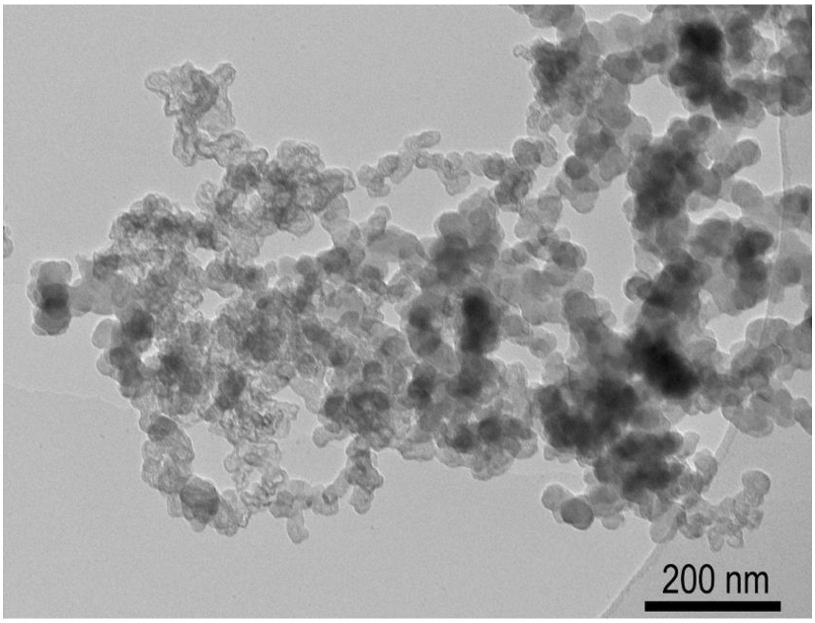

Supplementary Figure 5 | Morphology of Co-SAC. TEM image of Co-SAC.

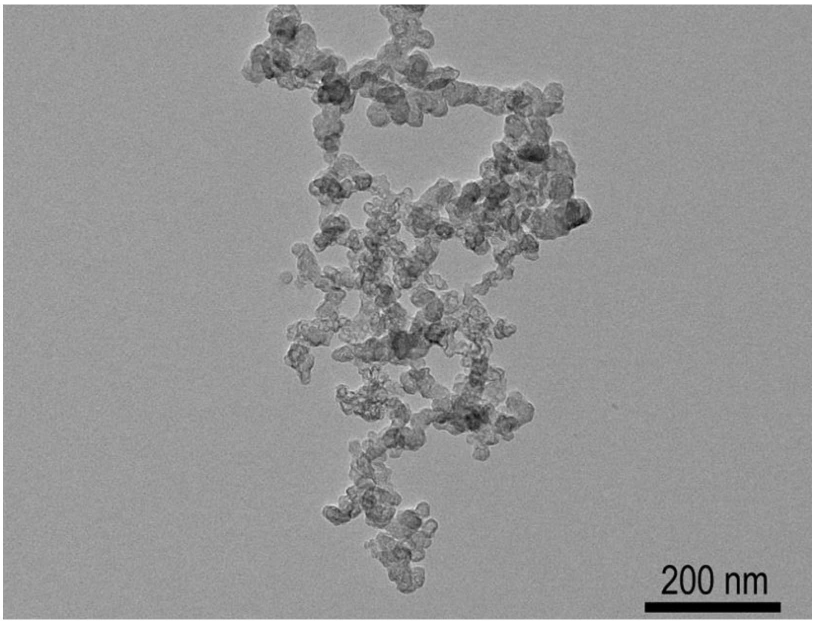

Supplementary Figure 6 | Morphology of Cu-SAC. TEM image of Cu-SAC.

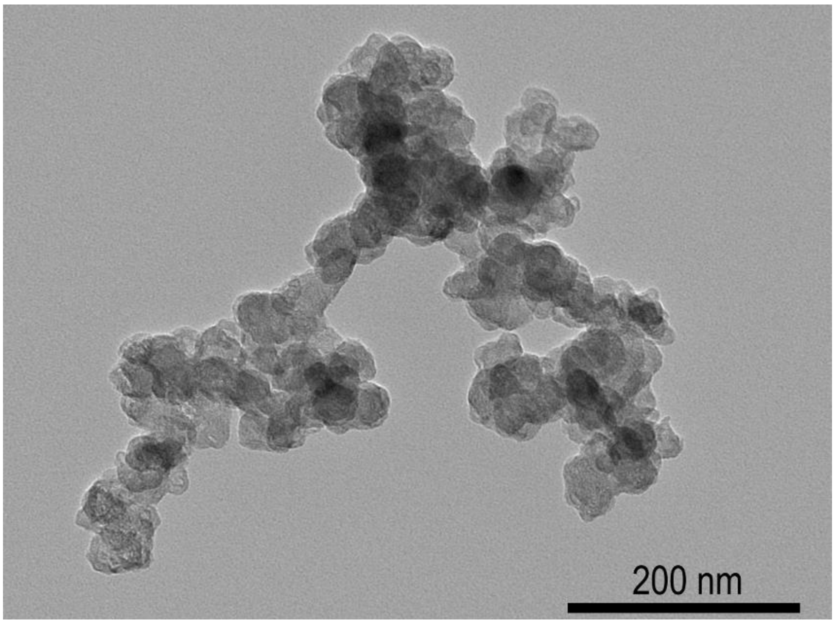

Supplementary Figure 7 | Morphology of Zn-SAC. TEM image of Zn-SAC.

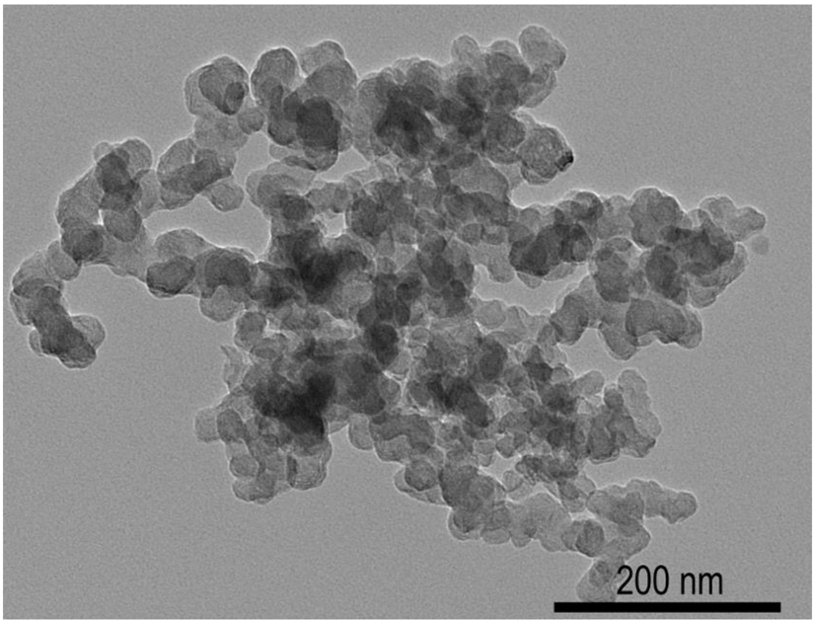

Supplementary Figure 8 | Morphology of Ru-SAC. TEM image of Ru-SAC.

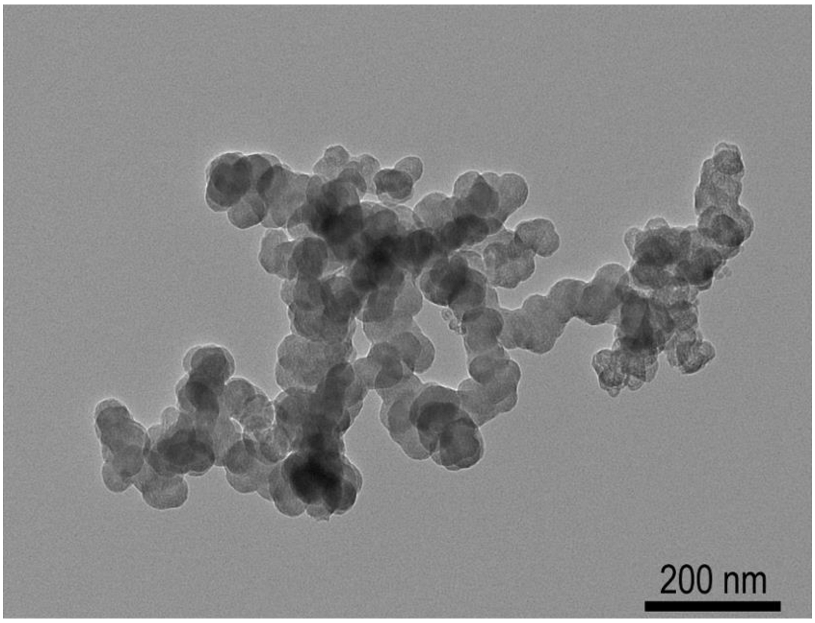

Supplementary Figure 9 | Morphology of Pt-SAC. TEM image of Pt-SAC.

Supplementary Figures 2-9 confirm that all M-SACs possessed the structure of carbon black. No metal nanoparticles were observed for any of the M-SACs, indicating that metal atoms in these materials were highly dispersed.

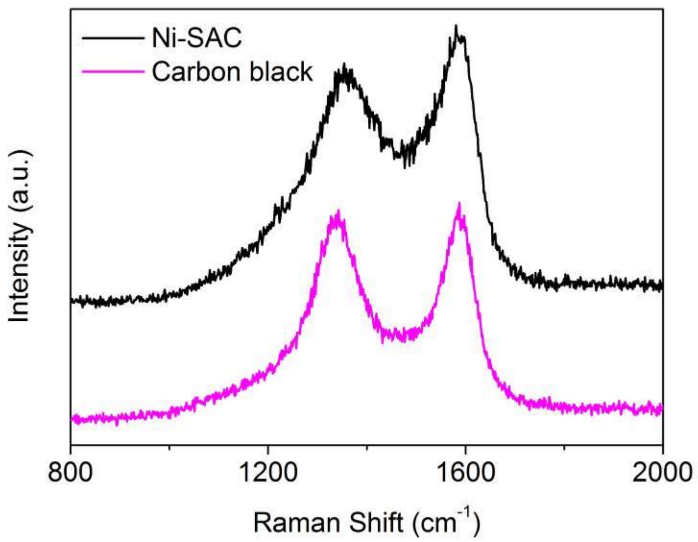

Supplementary Figure 10 | Characterization of Ni-SAC and carbon black. Raman spectra for Ni-SAC and carbon black.

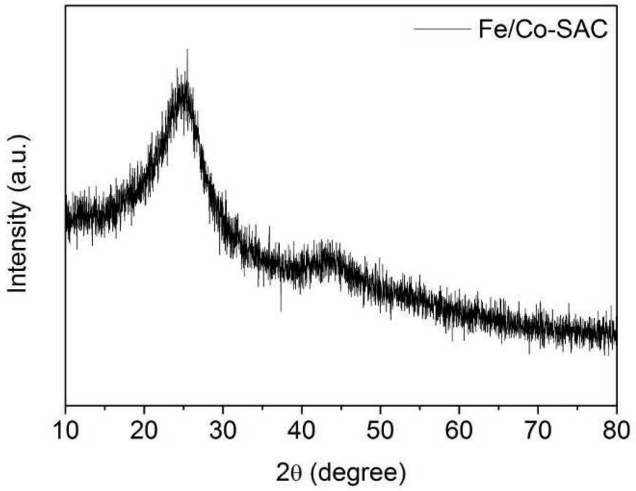

Supplementary Figure 11 | Characterization of a bimetallic SAC. XRD pattern for Fe/Co-SAC.

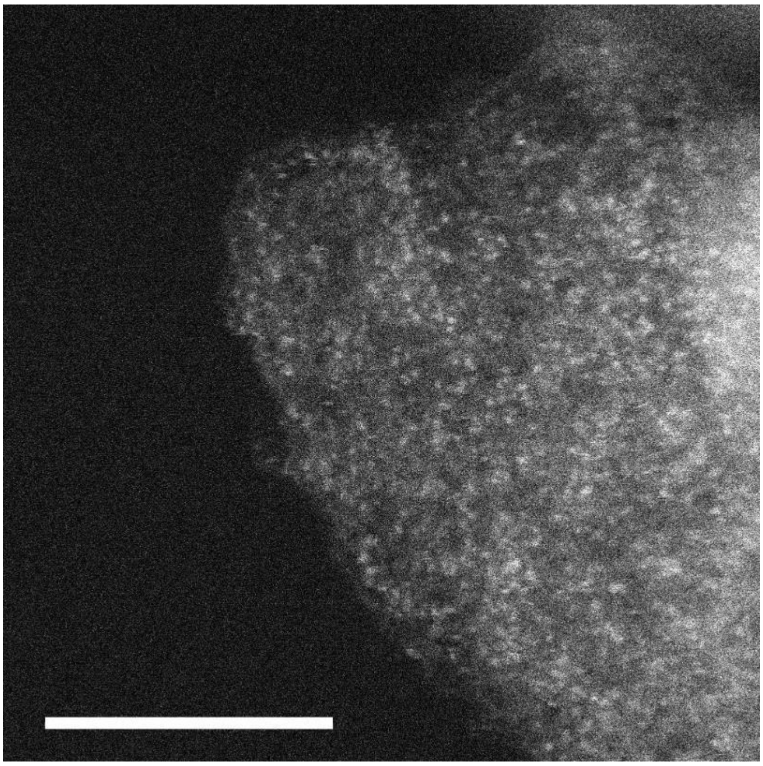

Supplementary Figure 12 | Morphology of a bimetallic SAC. HAADF-STEM image for a Fe/Co-SAC, scale bar: 5 nm.

Supplementary Figure 12 confirms that the Fe and Co were atomically dispersed in Fe/Co-SAC.

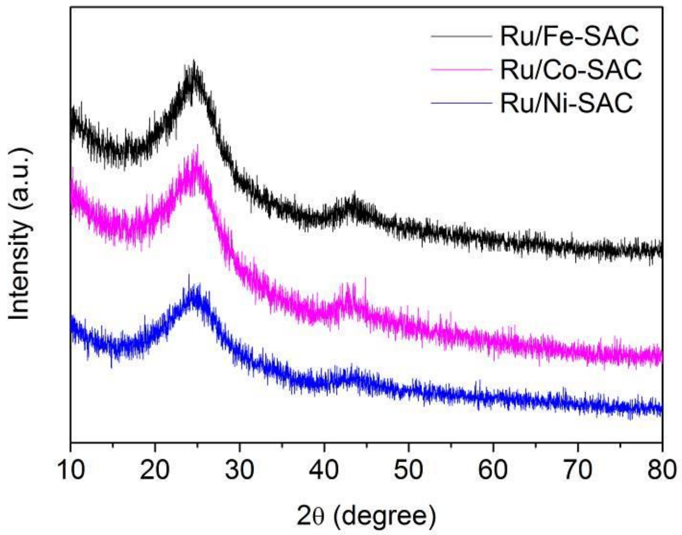

Supplementary Figure 13 | Characterization of bimetallic SACs. XRD patterns for Ru/Fe-SAC, Ru/Co-SAC, and Ru/Ni-SAC.

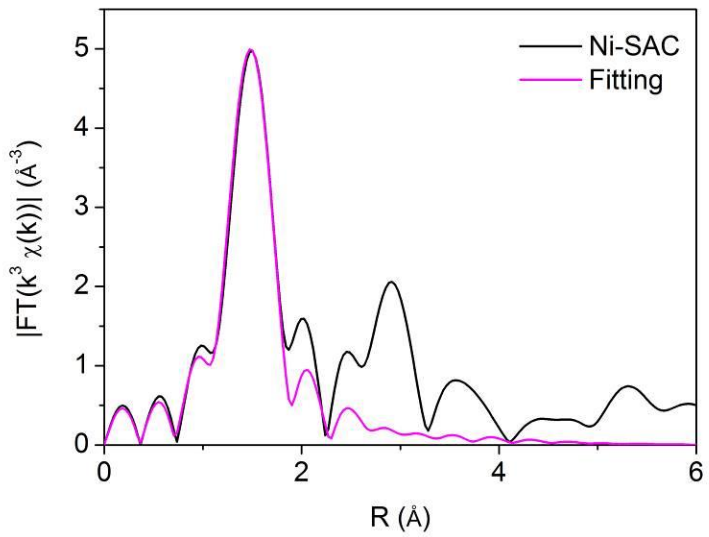

Supplementary Figure 14 | EXAFS fitting result for Ni-SAC. Ni K-edge EXAFS (R space plot) for Ni-SAC.

Supplementary Figure 14 confirms that Ni atoms in Ni-SAC were coordinated four fold by N atoms.

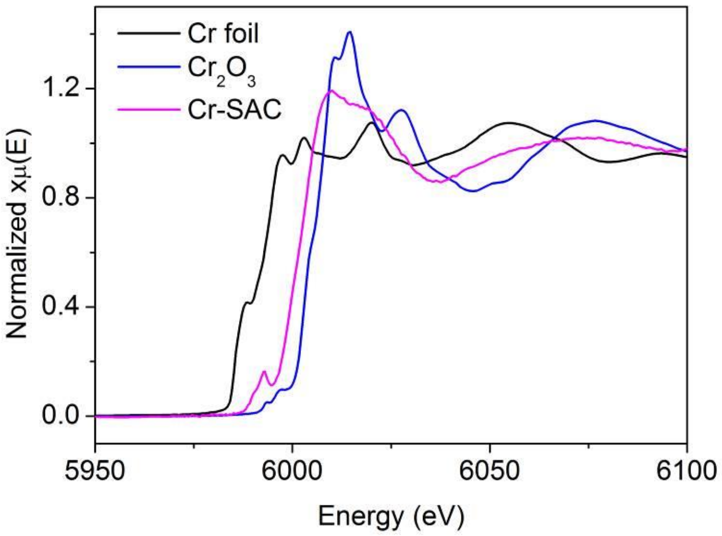

Supplementary Figure 15 | The oxidation state of Cr in Cr-SAC. Cr K-edge XANES spectra for Cr-SAC and different chromium reference materials.

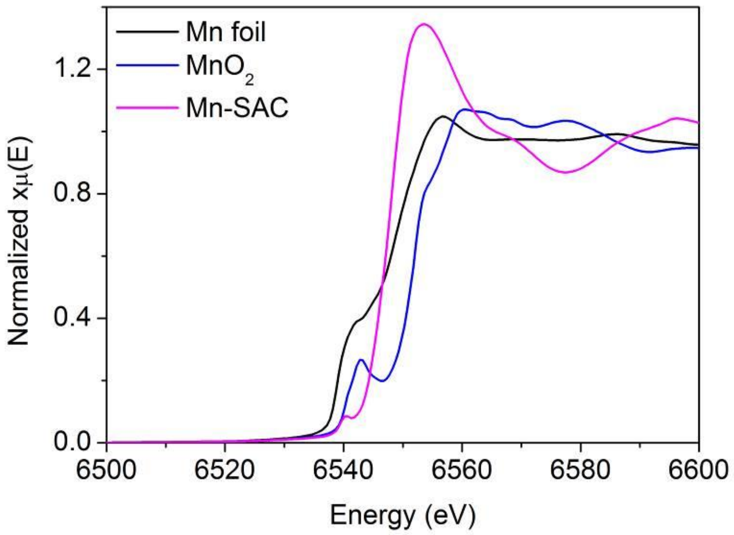

Supplementary Figure 16 | The oxidation state of Mn in Mn-SAC. Mn K-edge XANES spectra for Mn-SAC and a manganese foil reference.

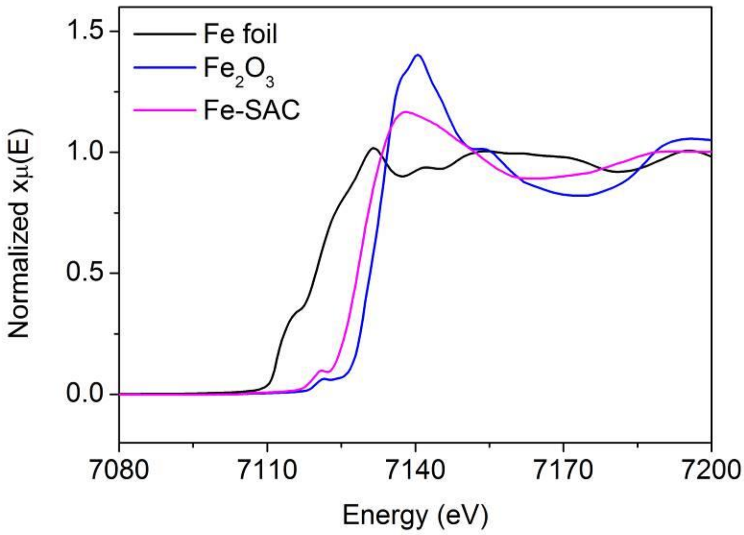

Supplementary Figure 17 | The oxidation state of Fe in Fe-SAC. Fe K-edge XANES spectra for Fe-SAC and different iron reference materials.

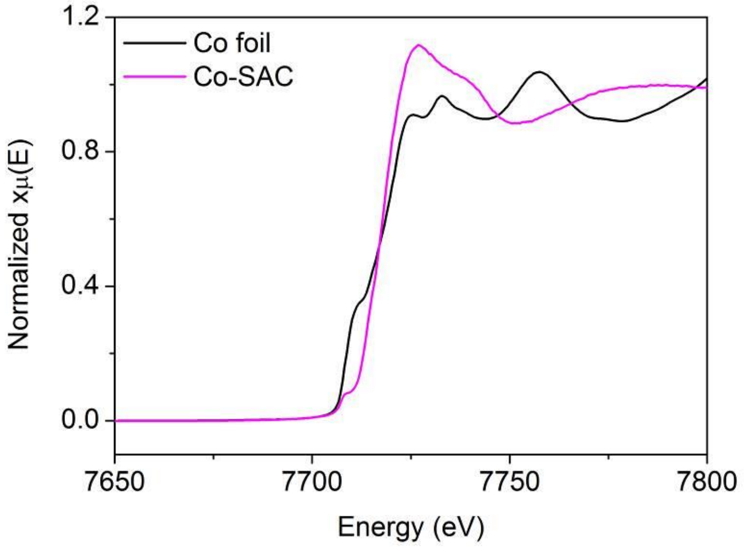

Supplementary Figure 18 | The oxidation state of Co in Co-SAC. Co K-edge XANES spectra for Co-SAC and a cobalt foil reference.

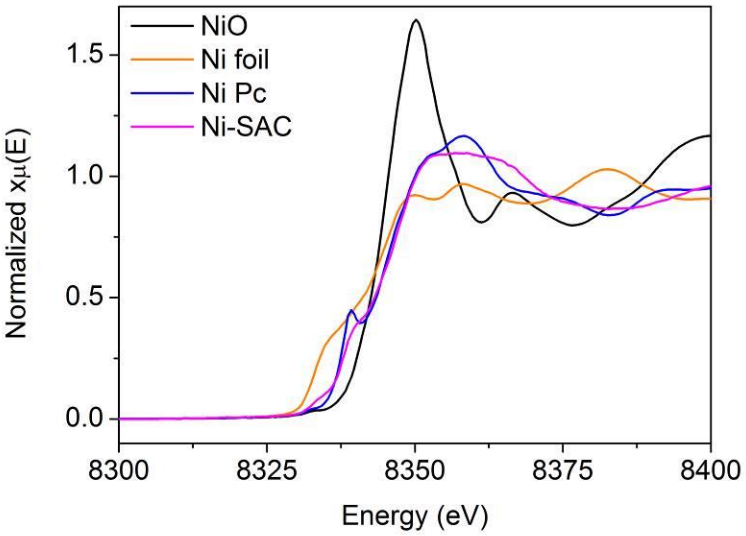

Supplementary Figure 19 | The oxidation state of Ni in Ni-SAC. Ni K-edge XANES spectra for Ni-SAC and different nickel reference materials. Ni-SAC was obtained by heating the Ni precursor (Ni Pc) at 600 ºC for 2 h under an Ar atmosphere.

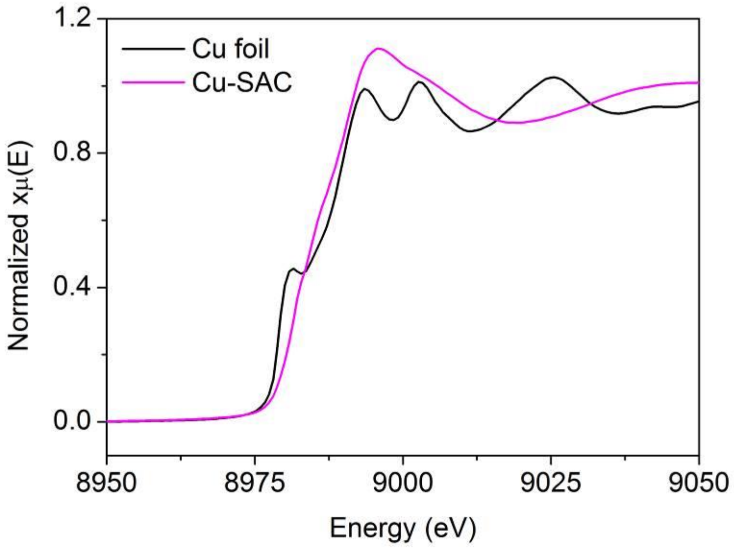

Supplementary Figure 20 | The oxidation state of Cu in Cu-SAC. Cu K-edge XANES spectra for Cu-SAC and a copper foil reference.

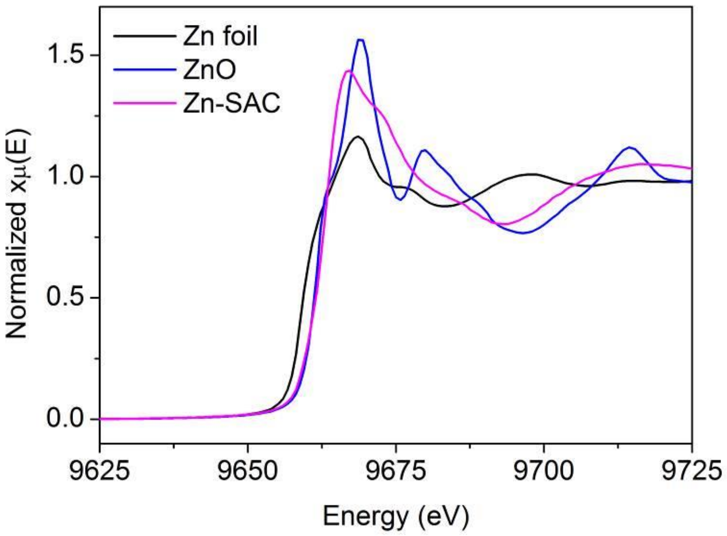

Supplementary Figure 21 | The oxidation state of Zn in Zn-SAC. Zn K-edge XANES spectra for Zn-SAC and different zinc reference materials.

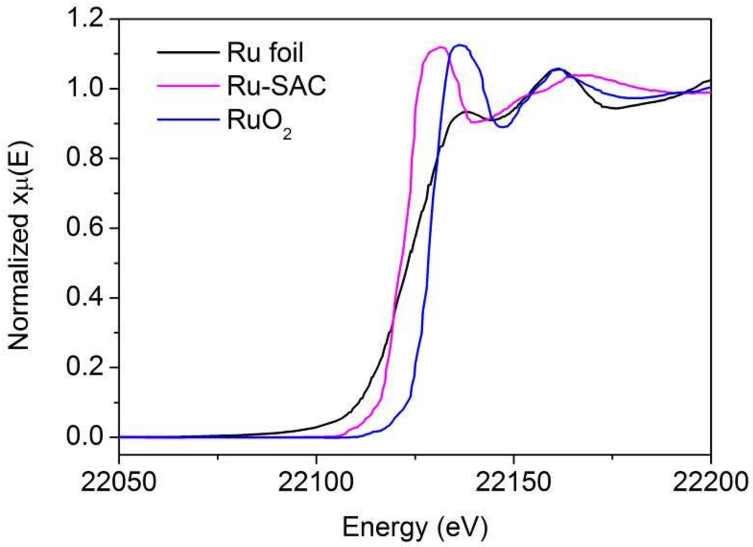

Supplementary Figure 22 | The oxidation state of Ru in Ru-SAC. Ru K-edge XANES spectra for Ru-SAC and different ruthenium reference materials.

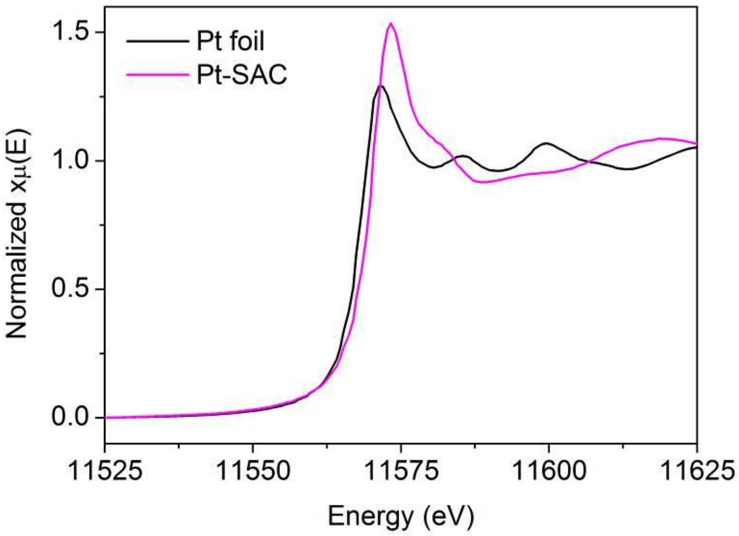

Supplementary Figure 23 | The oxidation state of Pt in Pt-SAC. Pt K-edge XANES spectra for Pt-SAC and a platinum foil reference.

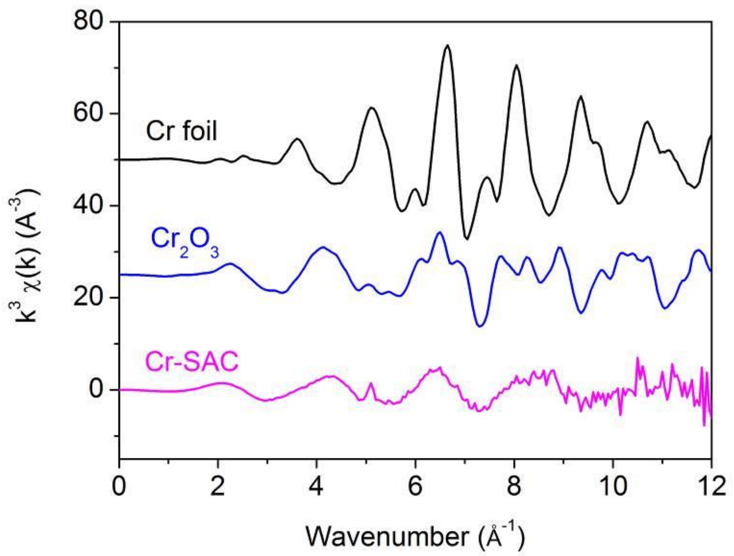

Supplementary Figure 24 | The structure of Cr-SAC. Cr K-edge EXAFS (k space plots) for Cr-SAC and different chromium reference materials.

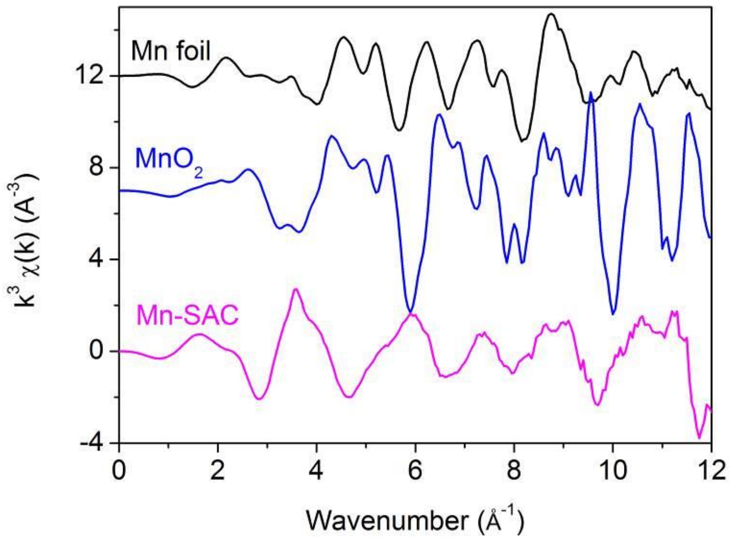

Supplementary Figure 25 | The structure of Mn-SAC. Mn K-edge EXAFS (k space plots) for Mn-SAC and a manganese foil reference.

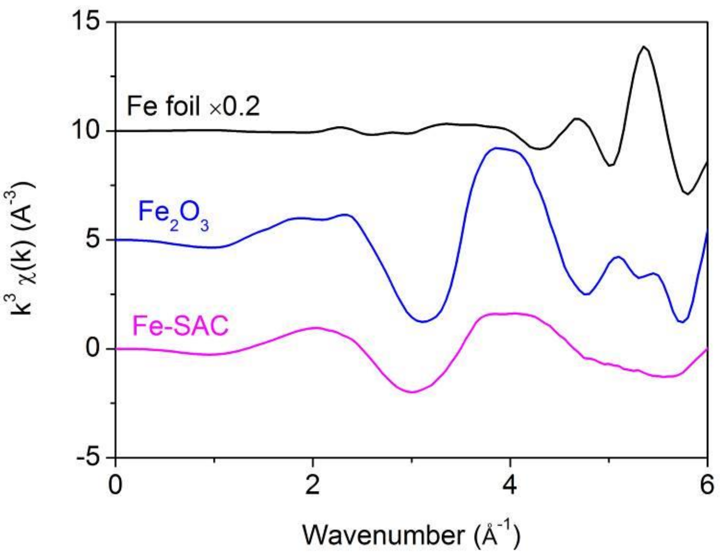

Supplementary Figure 26 | The structure of Fe-SAC. Fe K-edge EXAFS (k space plots) for Fe-SAC and different iron reference materials.

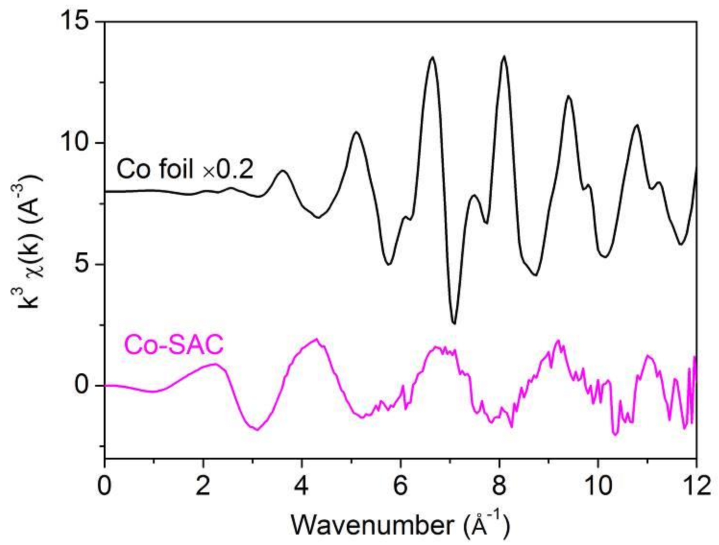

Supplementary Figure 27 | The structure of Co-SAC. Co K-edge EXAFS (k space plots) for Co-SAC and a cobalt foil reference.

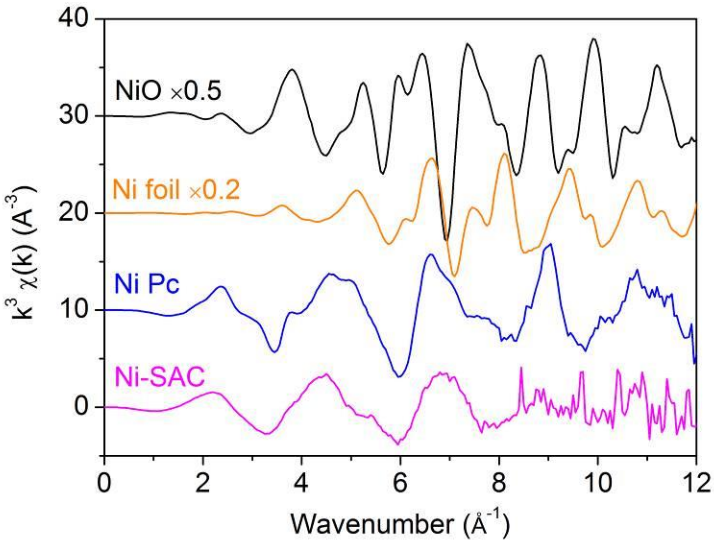

Supplementary Figure 28 | The structure of Ni-SAC. Ni K-edge EXAFS (k space plots) for Ni-SAC and different nickel reference materials

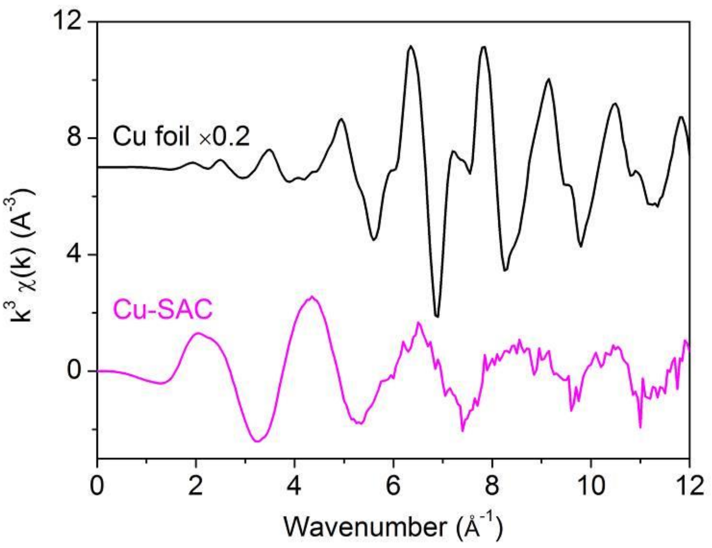

Supplementary Figure 29 | The structure of Cu-SAC. Cu K-edge EXAFS (k space plots) for Cu-SAC and a copper foil reference.

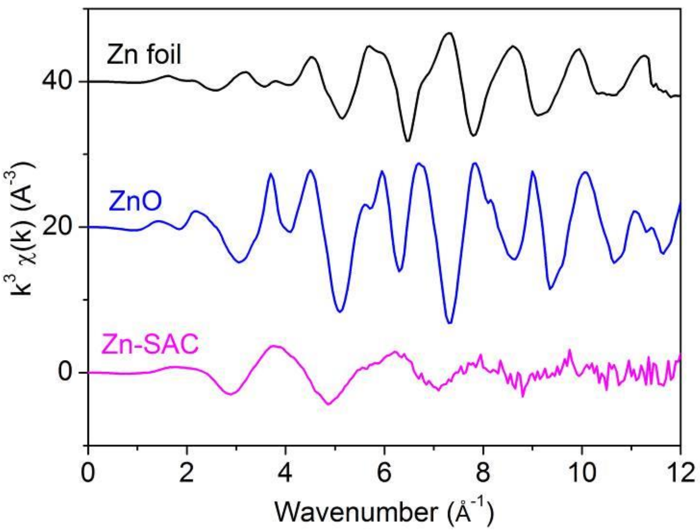

Supplementary Figure 30 | The structure of Zn-SAC. Zn K-edge EXAFS (k space plots) for Zn-SAC and different zinc reference materials.

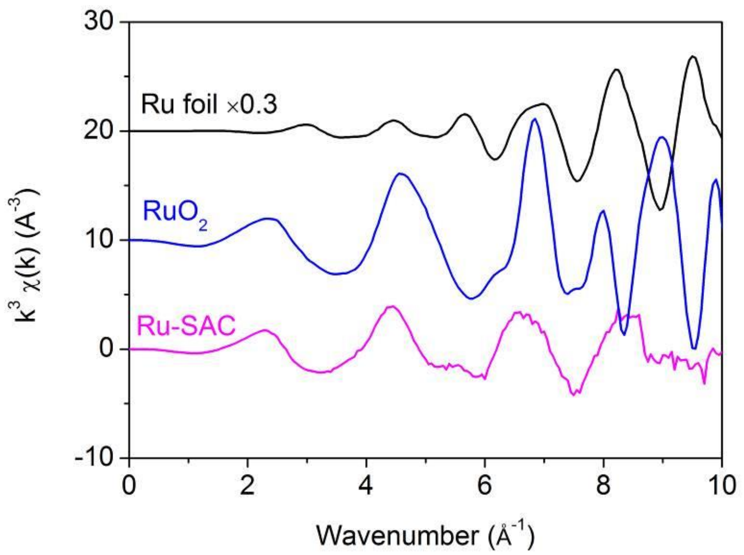

Supplementary Figure 31 | The structure of Ru-SAC. Ru K-edge EXAFS (k space plots) for Ru-SAC and different ruthenium reference materials.

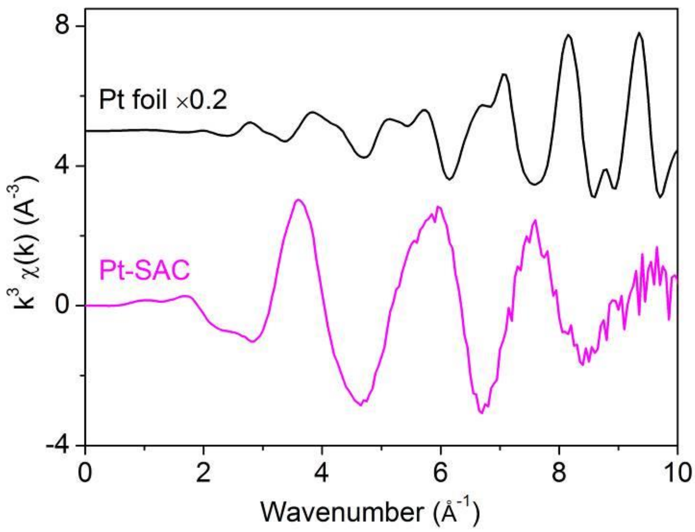

Supplementary Figure 32 | The structure of Pt-SAC. Pt K-edge EXAFS (k space plots) for Pt-SAC and a platinum foil reference.

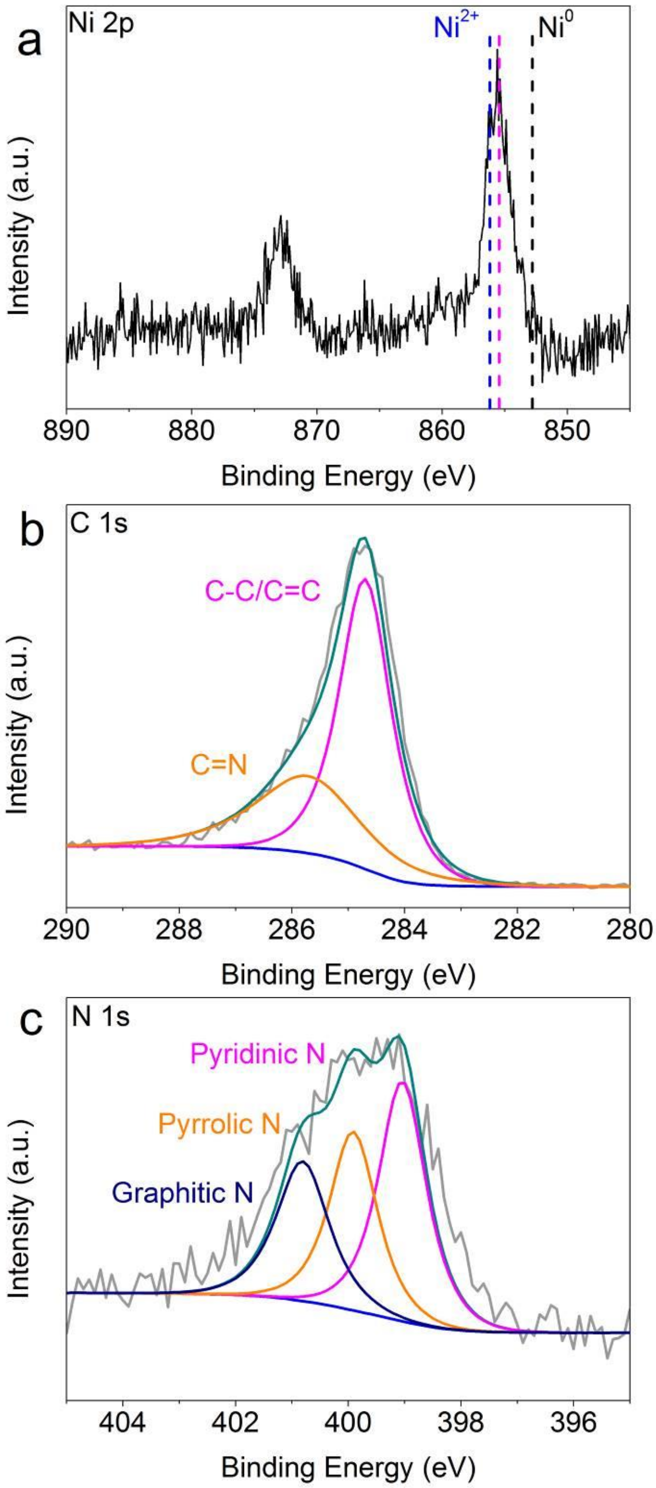

Supplementary Figure 33 | XPS spectra for Ni-SAC. a) Ni 2p region; b) C 1s region; c) N 1s region.

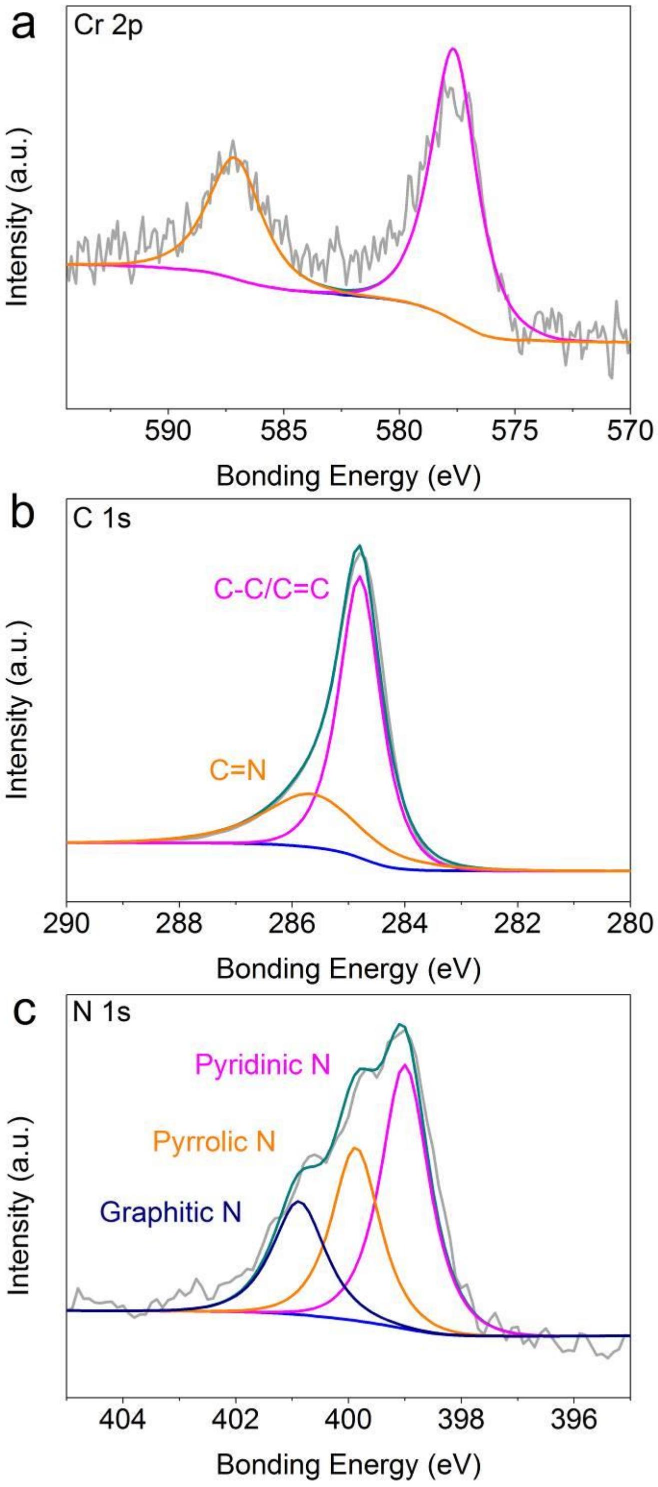

Supplementary Figure 34 | XPS spectra for Cr-SAC. a) Cr 2p region; b) C 1s region; c) N 1s region.

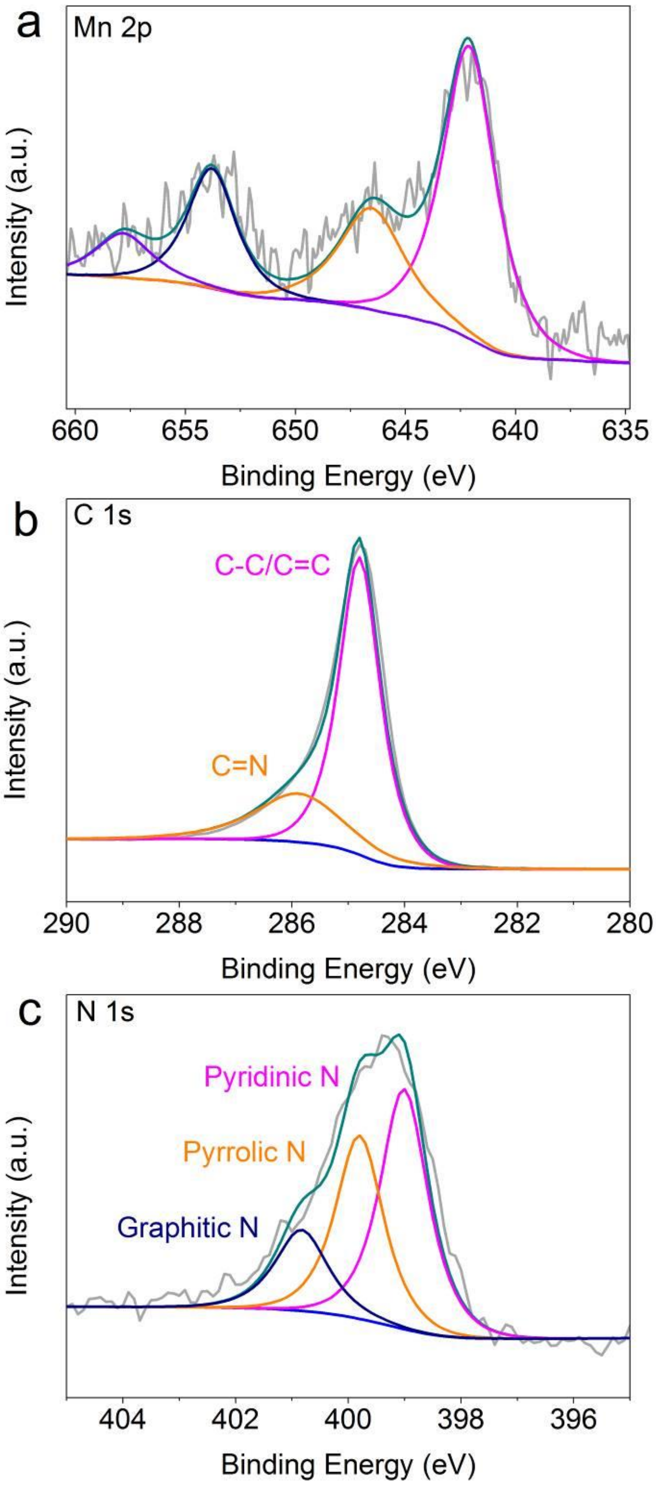

Supplementary Figure 35 | XPS spectra for Mn-SAC. a) Mn 2p region; b) C 1s region; c) N 1s region.

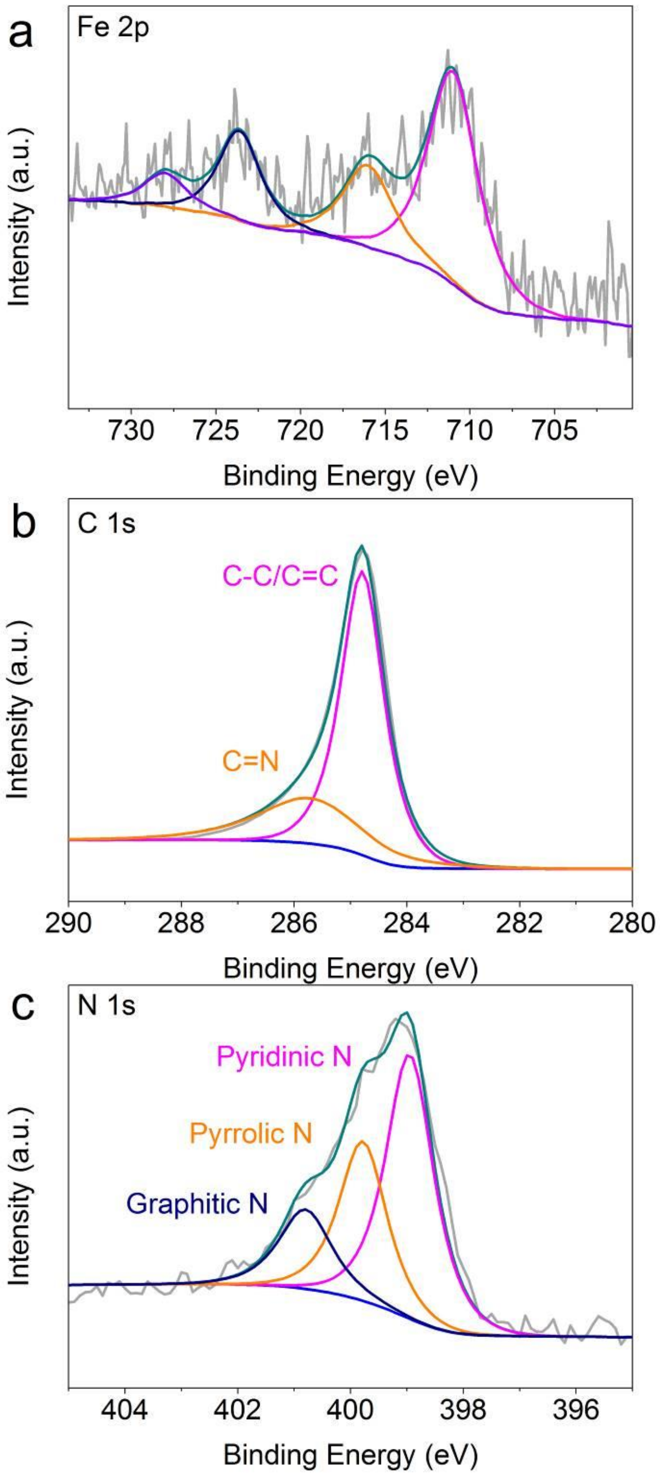

Supplementary Figure 36 | XPS spectra for Fe-SAC. a) Fe 2p region; b) C 1s region; c) N 1s region.

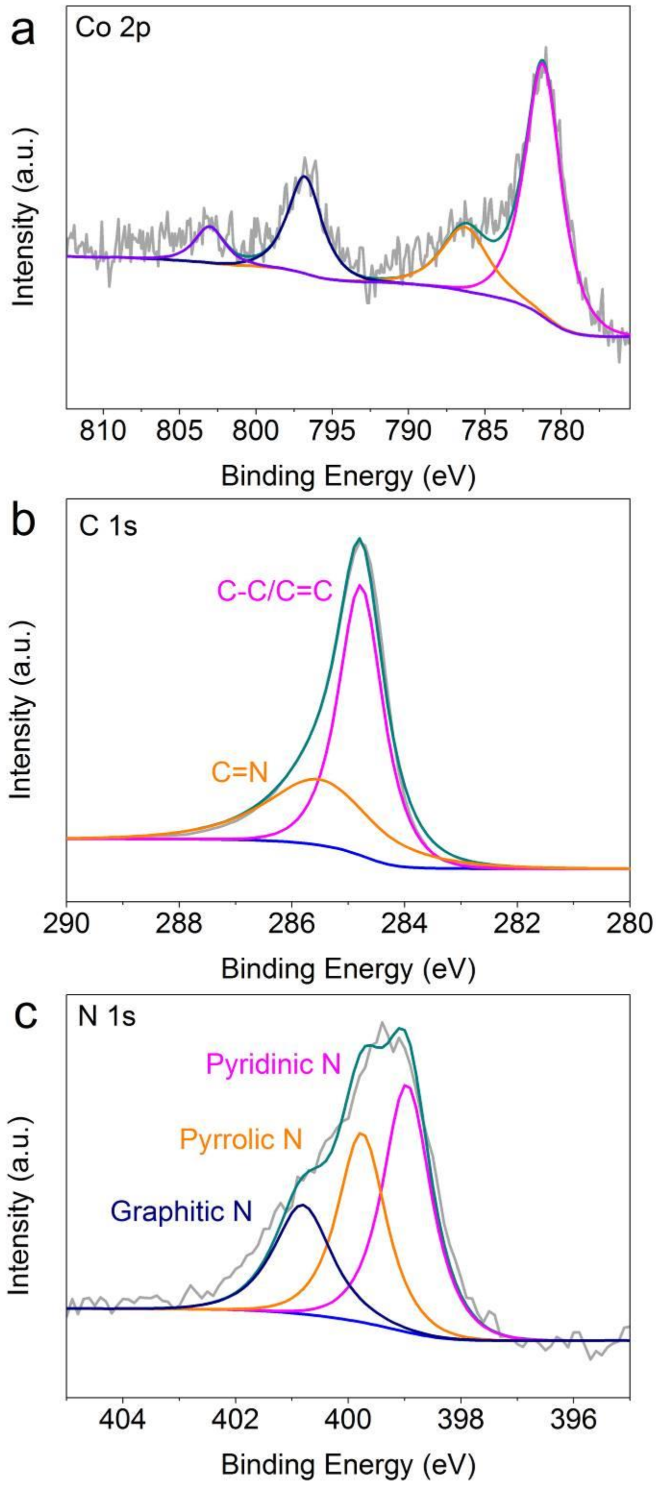

Supplementary Figure 37 | XPS spectra for Co-SAC. a) Co 2p region; b) C 1s region; c) N 1s region.

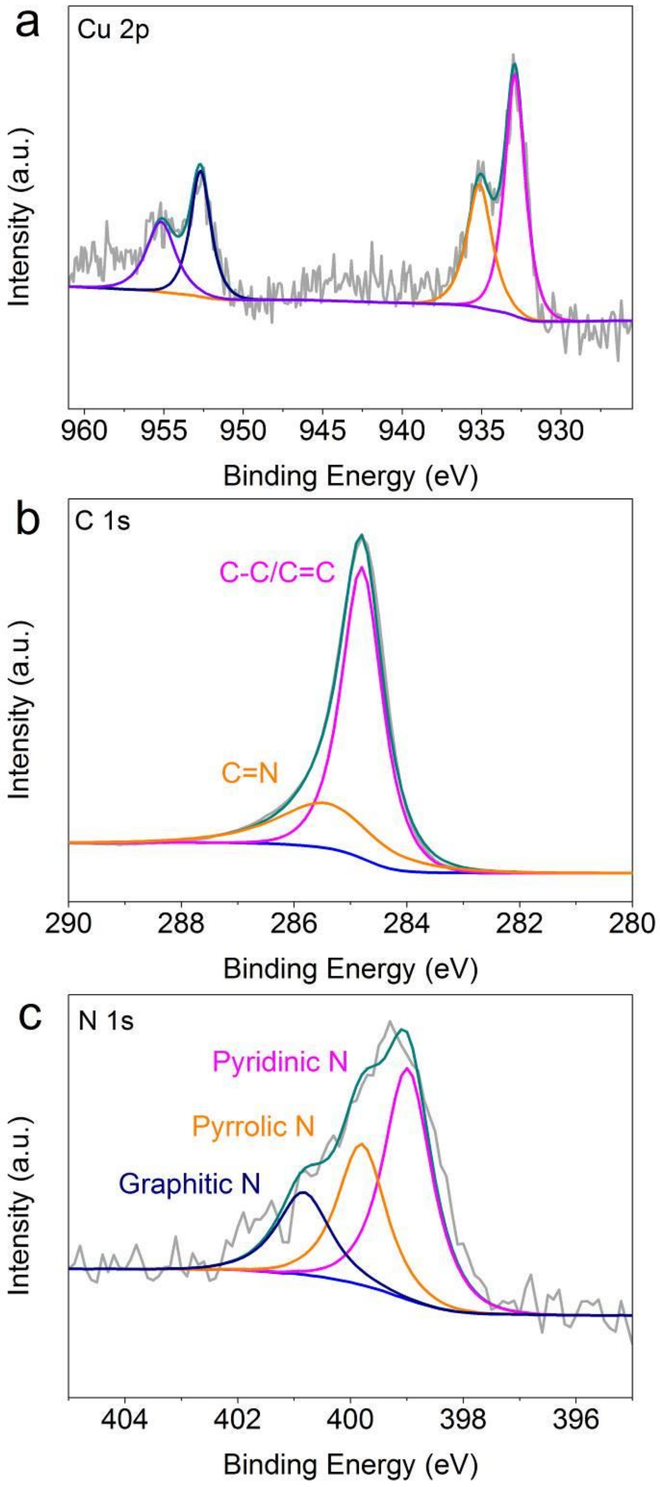

Supplementary Figure 38 | XPS spectra for Cu-SAC. a) Cu 2p region; b) C 1s region; c) N 1s region.

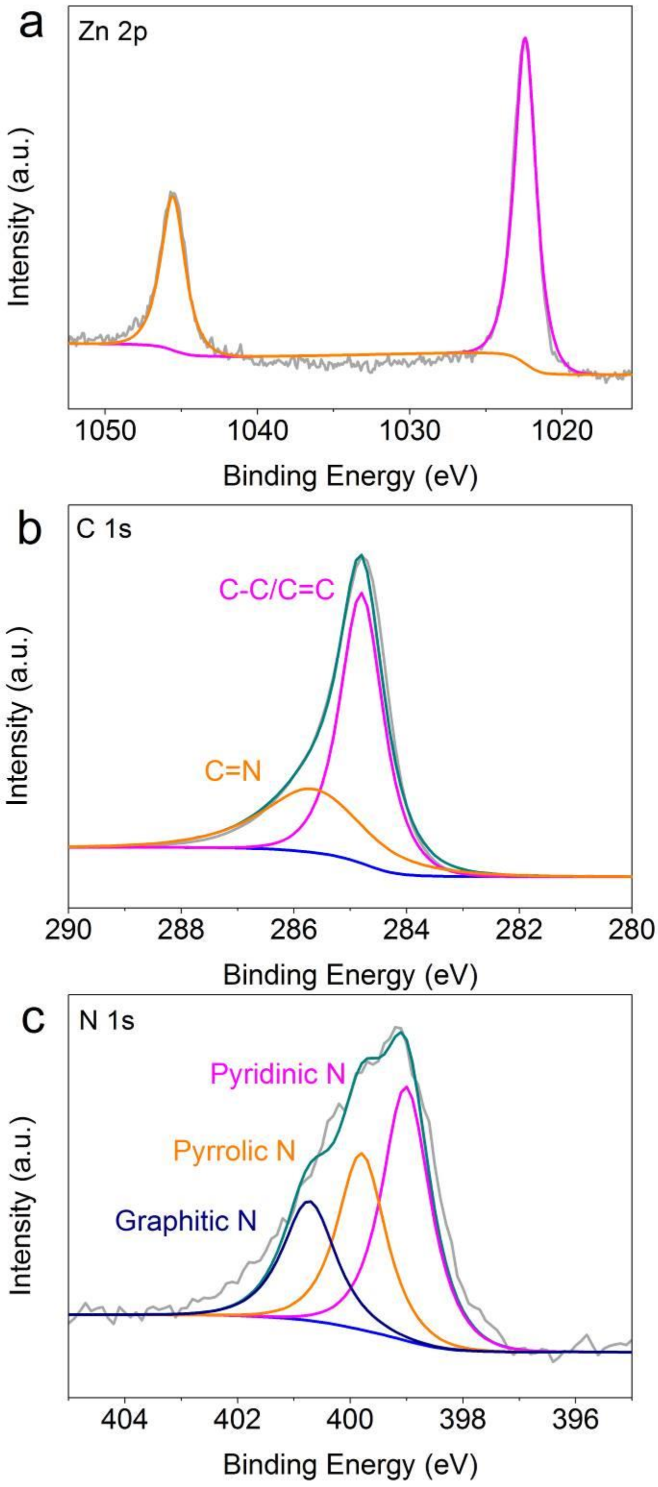

Supplementary Figure 39 | XPS spectra for Zn-SAC. a) Zn 2p region; b) C 1s region; c) N 1s region.

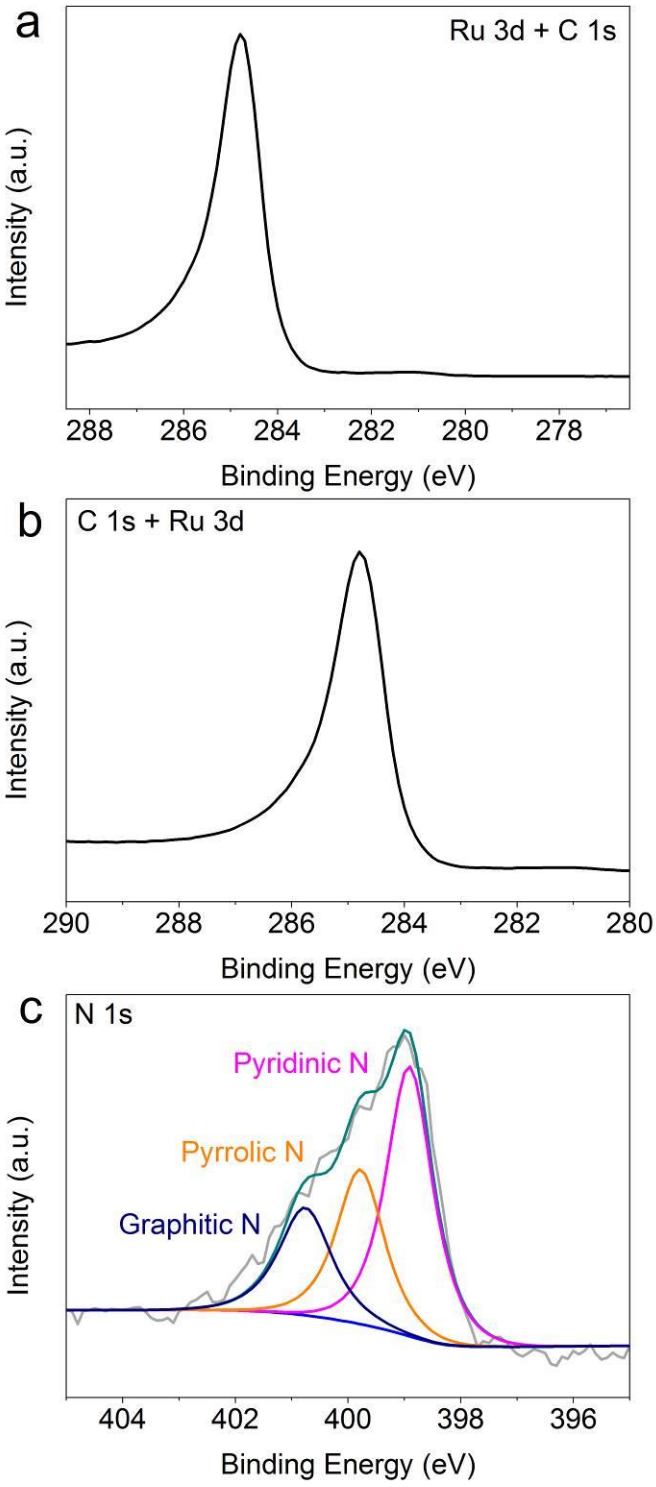

Supplementary Figure 40 | XPS spectra for Ru-SAC. a) Ru 3d region; b) C 1s region; c) N 1s region.

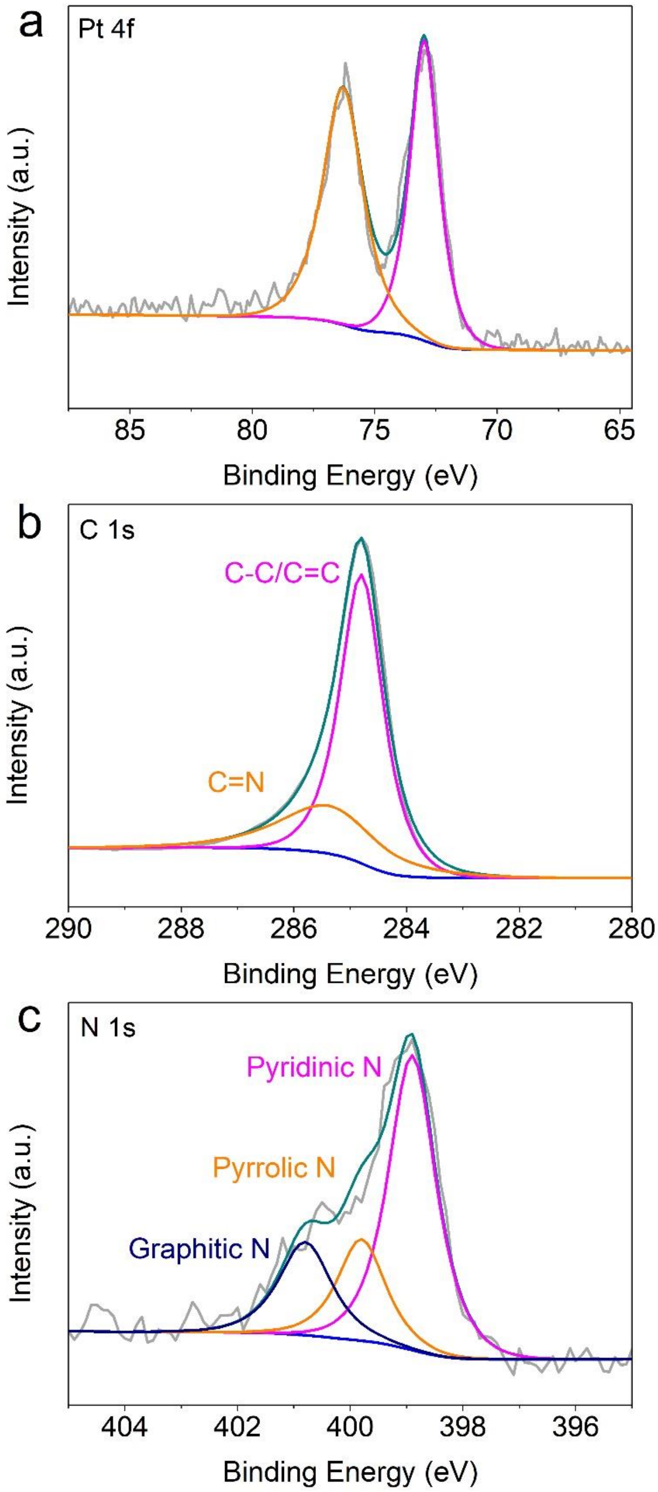

Supplementary Figure 41 | XPS spectra for Pt-SAC. a) Pt 4f region; b) C 1s region; c) N 1s region.

Supplementary Figure 33c indicates that the binding energy of the Ni $2p_{3/2}$ peak was 855.6 eV, higher than that observed for Ni^0 (852.8 eV)[1] and lower than that for Ni^{2+} in NiO (856.2 eV).[2] The data suggest the presence of Ni^{2+} in the N-SAC, with the lower binding energy reflecting Ni^{2+} bonding to less electronegative nitrogen rather than

oxygen. Supplementary Figures 33-41 confirm that all the other M-SACs contain M^{2+} cations. The C 1s spectra were deconvoluted into neutral carbon (i.e. C-C/C=C) and C-N species. The N 1s spectra were deconvoluted into pyridinic-N, pyrrolic-N, and graphitic-N species.[3]

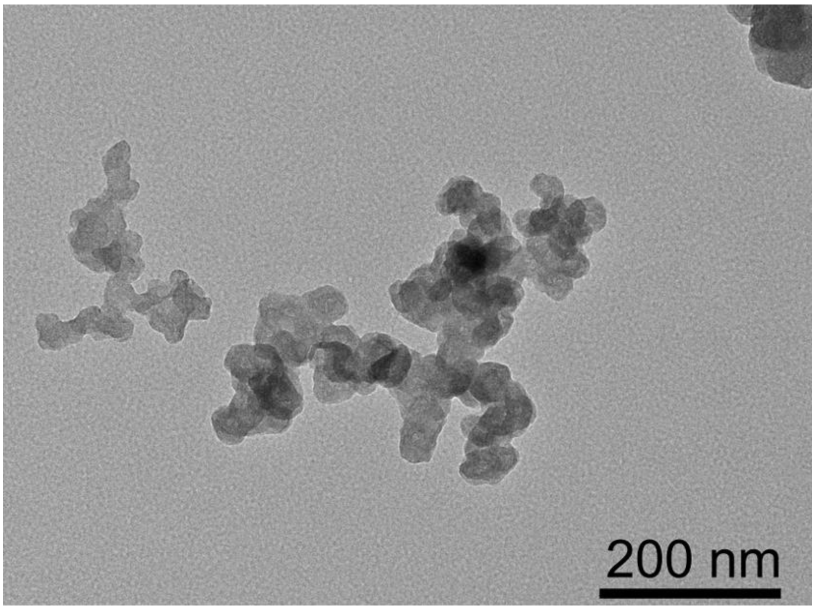

Supplementary Figure 42 | Morphology of Ni-SAC-2.5. TEM image of Ni-SAC-2.5.

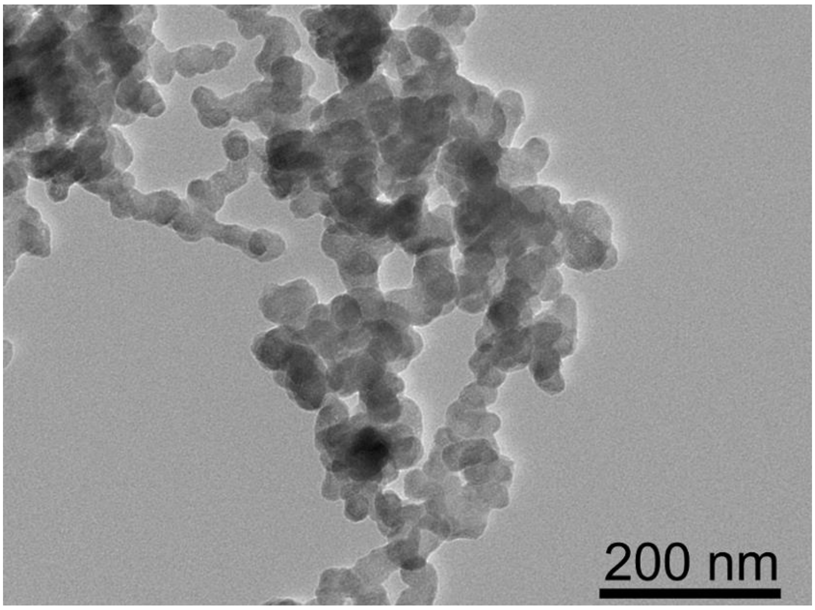

Supplementary Figure 43 | Morphology of Ni-SAC-3.4. TEM image of Ni-SAC-3.4.

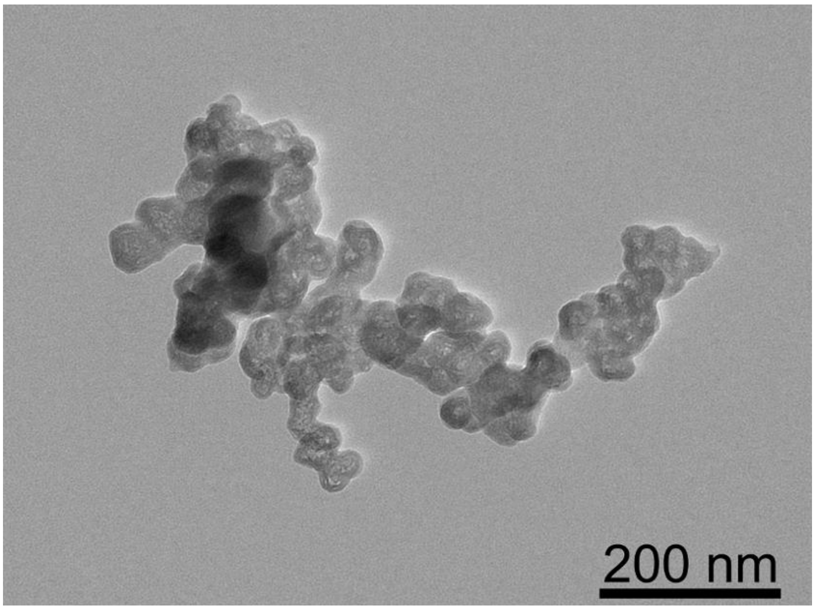

Supplementary Figure 44 | Morphology of Ni-SAC-4.5. TEM image of Ni-SAC-4.5.

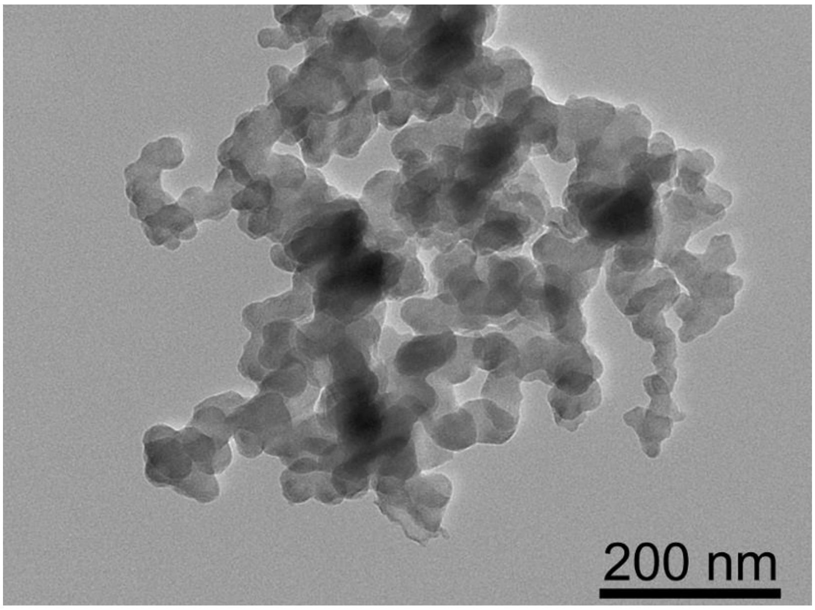

Supplementary Figure 45 | Morphology of Ni-SAC-5.3. TEM image of Ni-SAC-5.3.

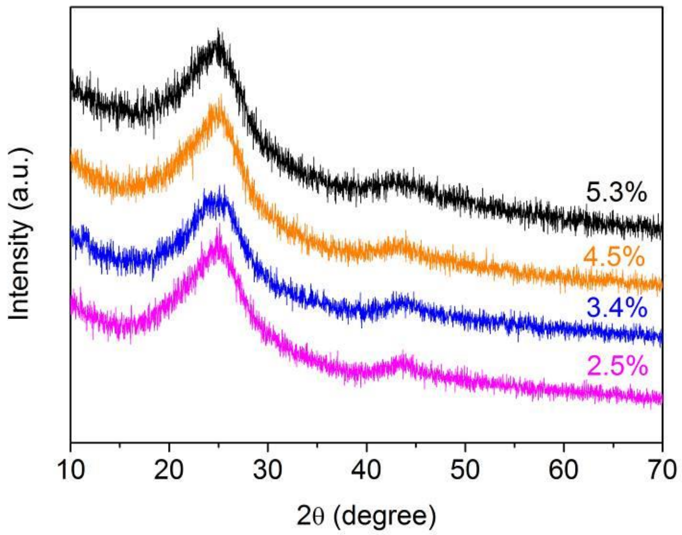

Supplementary Figure 46 | Characterization of Ni-SACs with different metal loadings. XRD patterns for Ni-SAC-*x* at different Ni loadings.

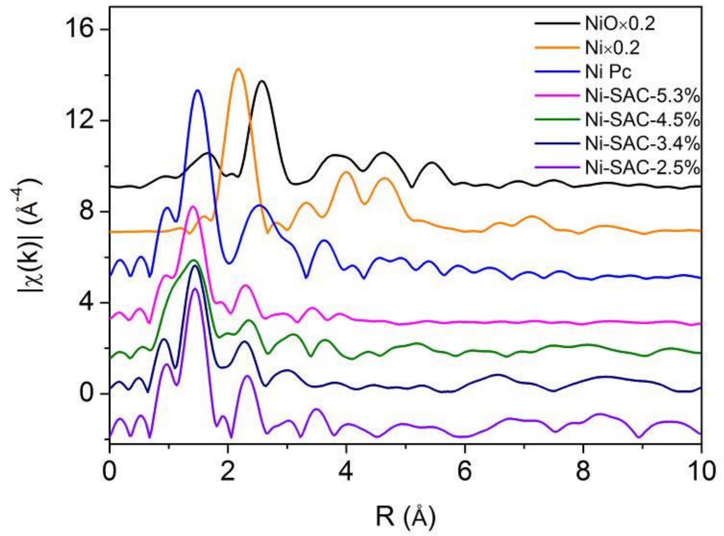

Supplementary Figure 47 | The structure of Ni-SACs with different metal loadings. Ni K-edge EXAFS (R space plots) for Ni-SAC-*x* at different Ni loadings.

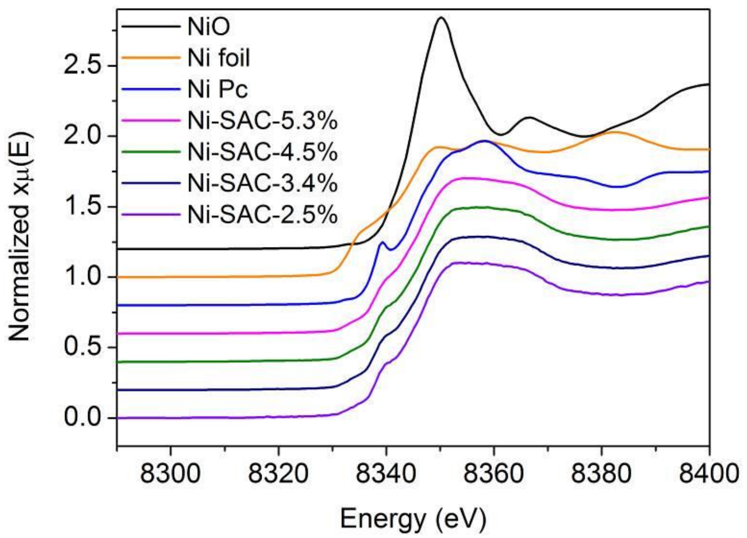

Supplementary Figure 48 | The oxidation state of Ni in Ni-SACs with different metal loadings. Ni K-edge XANES spectra for Ni-SAC-*x* with different Ni loadings.

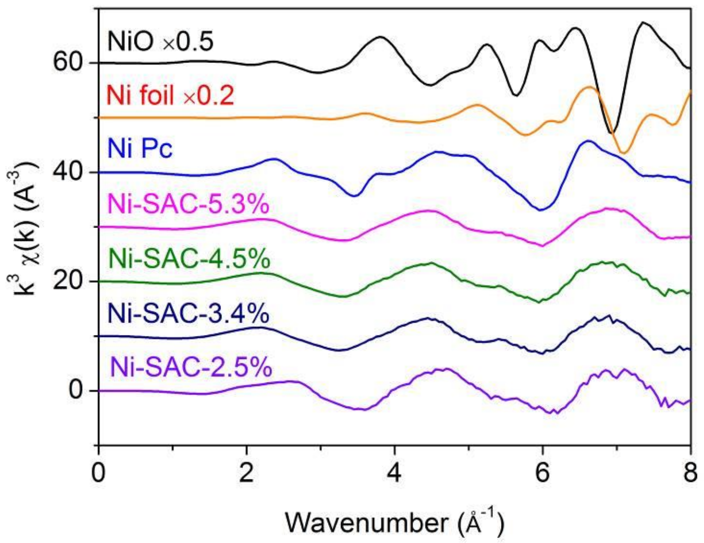

Supplementary Figure 49 | The structure of Ni-SACs with different metal loadings. Ni K-edge EXAFS (k space plots) for Ni-SAC-*x* at different Ni loadings.

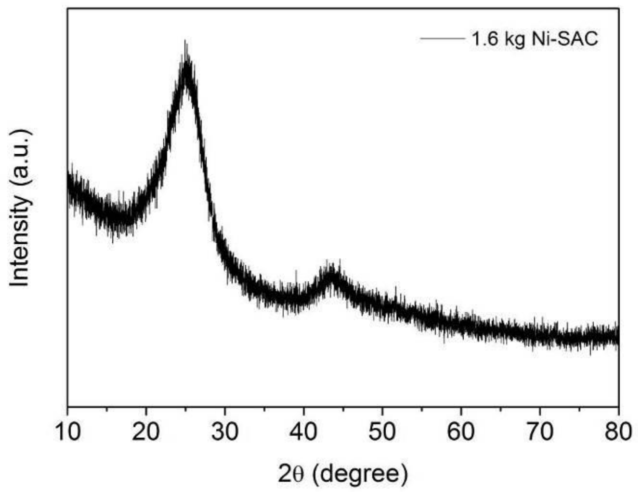

Supplementary Figure 50 | XRD pattern of Ni-SAC synthesized on a large scale.

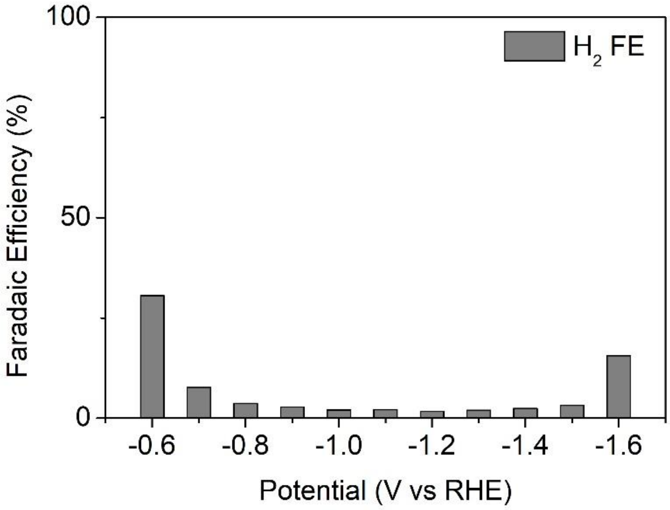

Supplementary Figure 51 | Faradaic efficiency of H_2 for Ni-SAC-2.5 at different potentials.

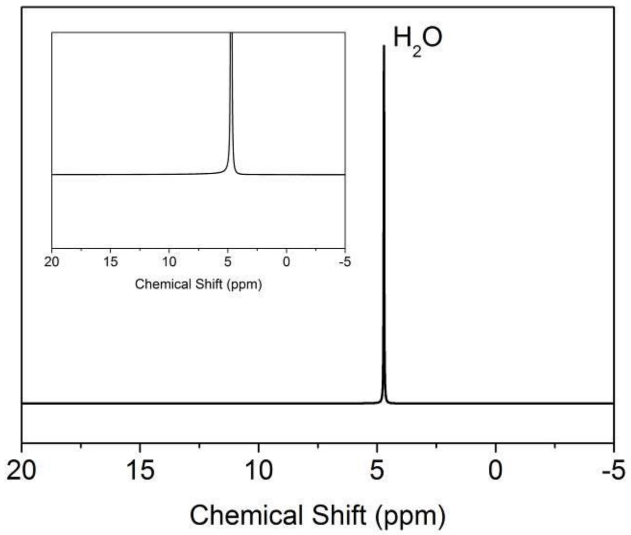

Supplementary Figure 52 | NMR spectrum of liquid products after CO_2RR. No liquid products were detected.

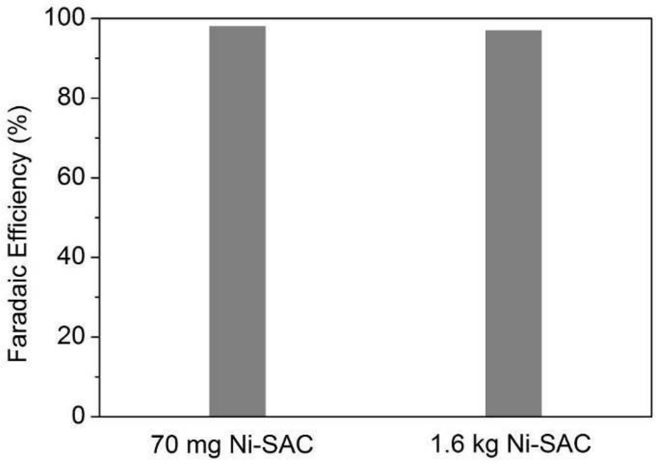

Supplementary Figure 53 | The CO_2RR performance of Ni-SAC synthesized on a large scale. CO_2RR activity of Ni SACs synthesized on a scale of 70 mg and on a scale of 1.6 kg.

Supplementary Figure 53 confirms that the Ni-SAC synthesized on a large scale (1.6 kg) had a very similar CO_2RR performance as Ni-SAC synthesized on a 70 mg scale, indicating that the samples were comparable in performance and that the universal M-SAC synthesis route introduced here was highly scalable.[4,5]

Supplementary Table 1 | Compositional analysis of the different M-SACs. The metal loading, total N content and N speciation of the different M-SACs is indicated.

**Sample**	**Metal loading wt.% (ICP)**	**Total N content at.% (XPS)**	**Pyridinic N content at.%**	**Pyrolic N content at.%**	**Graphitic N content at.%**
Cr-SAC	3.58	3.34	1.63	1.01	0.70
Mn-SAC	2.30	2.43	1.14	0.87	0.42
Fe-SAC	1.85	2.32	1.20	0.72	0.40
Co-SAC	2.97	3.26	1.42	1.09	0.75
Ni-SAC	5.32	6.75	3.42	1.93	1.40
Cu-SAC	2.59	3.4	1.71	0.87	0.82
Zn-SAC	3.65	3.55	1.61	1.11	0.83
Ru-SAC	2.67	2.12	1.04	0.61	0.47
Pt-SAC	2.16	1.57	0.93	0.32	0.32

Supplementary Table 2 | Metal loading comparison with other reported Ni-SAC materials

Sample	Metal loading	Measurement method	Reference
Ni-SAC@graphene	0.05 at.%	ICP	*6*
Ni-SAC@commercial carbon	0.27 wt.%	ICP	*7*
Ni-SAC@MOF	0.31 wt.%	ICP	*4*
Ni-SAC@g-C_3N_4	1.41 wt.%	ICP	*8*
Ni-SAC@MOF	1.53 wt.%	ICP	*5*
Ni-SAC@graphene oxide	0.44 at.%	XPS	*9*
Ni-SAC@g-C_3N_4	4.6 wt.%	ICP	*10*
Ni-SAC@commercial carbon	5.32 wt.%	ICP	**This work**

Supplementary References

1 Mansour, A. N. Nickel Monochromated Al Kα XPS Spectra from the Physical Electronics Model 5400 Spectrometer. *Surf. Sci. Spectra* **3**, 221-230 (1994).

2 Mansour, A. N. Characterization of NiO by XPS. *Surf. Sci. Spectra* **3**, 231-238 (1994).

3 Cao, Y., Mao, S., Li, M., Chen, Y. & Wang, Y. Metal/Porous Carbon Composites for Heterogeneous Catalysis: Old Catalysts with Improved Performance Promoted by N-Doping. *ACS Catal.* **7**, 8090-8112 (2017).

4 Yunteng Qu *et al.* Direct Transformation of Bulk Copper into Copper Single Sites via Emitting and Trapping of Atoms. *Nat. Catal.* **1**, 781-786 (2018).

5 Zhao, C. *et al.* Ionic Exchange of Metal-Organic Frameworks to Access Single Nickel Sites for Efficient Electroreduction of CO_2. *J. Am. Chem. Soc.* **139**, 8078-8081 (2017).

6 Fei, H. *et al.* General Synthesis and Definitive Structural Identification of MN_4C_4 Single-Atom Catalysts with Tunable Electrocatalytic Activities. *Nat. Catal.* **1**, 63-72 (2018).

7 Zheng, T. *et al.* Large-Scale and Highly Selective CO_2 Electrocatalytic Reduction on Nickel Single-Atom Catalyst. *Joule* **3**, 265-278 (2019).

8 Li, X. *et al.* Exclusive Ni-N_4 Sites Realize Near-Unity CO Selectivity for Electrochemical CO_2 Reduction. *J. Am. Chem. Soc.* **139**, 14889-14892 (2017).

9 Jiang, K. *et al.* Isolated Ni Single Atoms in Graphene Nanosheets for High-performance CO_2 Reduction. *Energy Environ. Sci.* **11**, 893-903 (2018).

10 Yang, H. B. *et al.* Atomically Dispersed Ni(i) as the Active Site for Electrochemical CO_2 Reduction. *Nat. Energy* **3**, 140-147 (2018).
